# Synergistic Effects of Atractylodes-Derived Sesquiterpenes and Polyacetylene on Chemotherapeutic Sensitivity in Cholangiocarcinoma: Impact on Transporter Gene Expression

**DOI:** 10.3390/molecules31071124

**Published:** 2026-03-29

**Authors:** Inthuon Kulma, Wanna Chaijaroenkul, Kesara Na Bangchang

**Affiliations:** 1 Graduate Program in Bioclinical Sciences, Chulabhorn International College of Medicine, Thammasat University (Rangsit Campus), Pathumthani 12120, Thailand; inthuorn.kulma@gmail.com (I.K.); wn_ap39@yahoo.com (W.C.); 2 Center of Excellence in Pharmacology and Molecular Biology of Malaria and Cholangiocarcinoma, Thammasat University (Rangsit Campus), Pathumthani 12120, Thailand; 3 Drug Discovery and Development Center, Office of Advanced Science and Technology, Thammasat University (Rangsit Campus), Pathumthani 12120, Thailand

**Keywords:** atractylodin, β-eudesmol, chemotherapeutic drug, drug transporters, cholangiocarcinoma, gene expression

## Abstract

*Atractylodes lancea* (AL) has been shown to be a promising candidate for the treatment of cholangiocarcinoma (CCA). The study explored the potential of atractylodin (AT) and β-eudesmol (BE) to chemosensitize the effects of standard chemotherapeutics in CCA. The cytotoxicities of AT and BE on CL6, HuCCT1, and HuH28 when used in combination with 5-fluorouracil (5FU), gemcitabine (GEM), and cisplatin (Cis) were assessed by MTT assay. The modulatory effects of both compounds on mRNA expression of the reuptake and efflux transporters were determined by real-time PCR. The FIC (Fractional Inhibitory Concentration) indices indicated synergistic interactions (AT-5FU in all cell lines and BE-5FU in HuH28) and antagonistic interactions (BE-Cis in all cell lines and AT-Cis or AT-GEM in HuCCT1). The synergistic interactions observed with the AT-5FU and BE-5FU combinations were well correlated with the significant upregulation of the mRNA expression of the reuptake transporter genes *hENT1* (2.64-fold) and *hOCT3* (5.02-fold) and the significant downregulation of the mRNA expression of the efflux transporter gene *ABCC2* (0.33-fold). AT and BE, when purified or present as significant components in AL, may benefit CCA treatment when used as adjunct therapy to standard chemotherapeutic drugs, particularly 5FU. The mechanism of synergistic activity may, at least in part, involve modulation of transporter gene expression and activity.

## 1. Introduction

Cholangiocarcinoma (CCA) is a type of cancer that forms in the bile duct. Although the prevalence of CCA is low worldwide, marked increases in prevalence and mortality have been reported, particularly in northeastern Thailand [1,2,3]. Key risk factors include chronic biliary inflammation, liver fluke infection (*Opisthorchis viverrini* and *Clonorchis sinensis*), primary sclerosing cholangitis, hepatolithiasis, and viral hepatitis. The regional burden in Thailand has been strongly linked to chronic infection with the liver fluke *O. viverrini*, a well-established risk factor that promotes persistent biliary inflammation and carcinogenesis. Surgical resection of the tumour is the first-line treatment for CCA, but it is only effective in early-stage disease [4]. Moreover, only 25% of CCA patients are resectable at diagnosis [5]. As the clinical presentation of CCA is non-specific [6], diagnosis in early-stage CCA is difficult, and most patients have a poor prognosis [7]. Chemotherapy is recommended to prolong the survival rate in unresectable or advanced-stage CCA patients [8]. Gemcitabine alone or in gemcitabine-based regimens is recommended for these patient groups [9]. In recent years, advances in molecular profiling have led to the development of targeted therapies for specific genetic alterations in CCA. For example, inhibitors targeting fibroblast growth factor receptor 2 (FGFR2) fusions, such as pemigatinib and futibatinib, have shown promising clinical activity in patients with previously treated advanced cholangiocarcinoma harboring FGFR2 alterations. Furthermore, the isocitrate dehydrogenase 1 (IDH1) inhibitor ivosidenib has been approved for patients with IDH1-mutated CCA [10].

Cisplatin is known to have additive or synergistic effects when combined with gemcitabine across several tumour types, including lung [11], bladder [12], and head and neck cancers [13]. Gemcitabine is a nucleoside analogue that exerts its anticancer activity by inhibiting DNA synthesis after intracellular phosphorylation to its active metabolites, which are incorporated into DNA, leading to chain termination and apoptosis. Cisplatin, in contrast, acts primarily by forming DNA crosslinks, resulting in DNA damage and the activation of cell death pathways. These mechanisms contribute to their widespread use as first-line chemotherapeutic agents in advanced CCA [14,15,16]. In CCA, previous studies have shown that combination therapy with gemcitabine and cisplatin yields longer survival and a higher response rate than monotherapy [14,15,16]. However, a major problem with CCA chemotherapy is the development of chemoresistance, particularly multidrug resistance (MDR). Several molecular mechanisms underlying anti-CCA drug resistance have been reported, including alterations in drug transport mediated by ATP-binding cassette (ABC) transporters, such as multidrug resistance protein 1 (*MDR1*/P-Gp, *ABCB1*) and multidrug resistance-associated protein 1 (*MRP1/ABCC1*), which enhance drug efflux and decrease intracellular drug accumulation [1]. The gemcitabine-resistant cell lines KKU-M139/GEM and KKU-M214/GEM, both of which are resistant sublines, exhibited cross-resistance to several anticancer agents, including 5FU, doxorubicin, and paclitaxel, by upregulating *MRP1* and downregulating *hENT1* [2,17]. Nucleoside analogs such as gemcitabine and 5-fluorouracil are taken up by the Equilibrative Nucleoside Transporters (ENTs) and the Concentrative Nucleoside Transporters (CNTs) [18]. The Organic Cation Transporters (OCTs) are involved in the cellular uptake of platinum drugs such as cisplatin [19]. Downregulation of expression of the reuptake transporters *SLC22A1* (*hOCT1*), *SLC22A3* (*hOCT3*) [20], and *SLC29A1* (*hENT1*) [21], and upregulation of expression of the efflux transporters *ABCC1* (*MRP1*) [22] and *ABCB1* (*MDR1*) [8], were reported in CCA. Furthermore, various CCA cell lines reported a strong correlation between *ABCC3* (*MRP3*) expression and multidrug resistance [8].

**Figure 1:**
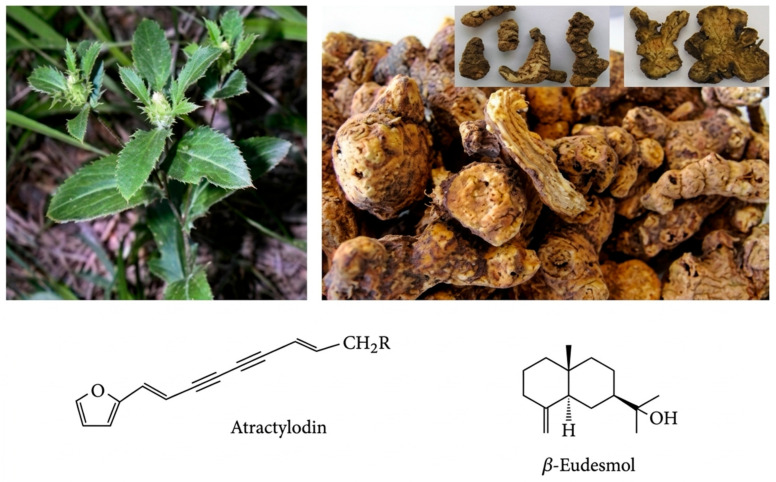
Chemical structures of the major bioactive compounds from *Atractylodes lancea*: Atractylodin (polyacetylene compound) and β-Eudesmol (sesquiterpenoid alcohol) [35].

The dried rhizome of *Atractylodes lancea* (Thunb.) DC. (AL) is well-known and widely used in traditional medicine as “Mao Cangzhu” in China [23], “So-Jutsu” in Japan [24], and Khod-Kha-Mao” in Thailand. Modern pharmacological studies show that AL broadly affects the nervous, gastrointestinal, and cardiovascular systems [25]. The crude extract of the AL rhizome has been shown to possess anti-gastric cancer, anti-gastric ulcer, and anti-inflammatory activities [26,27,28]. Primary active ingredients in AL include sesquiterpenoids (atractylon, β-eudesmol, and hinesol) and polyacetylene (atractylodin) [25]. The anti-CCA potential of the two major bioactive compounds, atractylodin and β-eudesmol (Figure 1), was demonstrated in the human CCA cell line CL-6 [29,30], including inhibition of cell growth, promotion of cell cycle arrest, induction of apoptosis, and effects on cell survival and migration. Interestingly, a recent study showed that β-eudesmol could improve the cytotoxic effect of 5-fluorouracil (5FU) and doxorubicin in the human CCA cell line KKU-100, along with suppression of the expression and activity of NAD(P)H-quinone oxidoreductase-1 (NQO1) enzyme, and inhibition of cell migration through induction of cell apoptosis and activation of caspase 3/7 [31]. In addition, the expression of ATP-binding cassette sub-family A member 12 (*ABCA12*) and the ATP-binding cassette sub-family G member 8 (*ABCG8*) was downregulated in CL-6 cells after 48 h of exposure to atractylodin [32]. It is possible that atractylodin and β-eudesmol could significantly enhance the cytotoxic effects of conventional chemotherapeutic drugs in CCA cells by modulating transporter gene expression. As AL is under research and development for CCA, it is necessary to investigate the anti-CCA interactions between its various chemical components. A Phase I clinical trial demonstrated a satisfactory safety and tolerability profile for the standardized AL rhizome extract capsule formulation [33]. The Phase II clinical trial [34] and preliminary results show remission of anti-CCA activity in AL in patients with advanced-stage CCA. AL will likely be used as adjunctive therapy to improve the clinical efficacy and tolerability of standard regimens for treating CCA.

The present study aimed to explore the possible roles of the two major bioactive compounds atractylodin and β-eudesmol in sensitizing the cytotoxic activities of gemcitabine, cisplatin, and 5FU in CCA. Association between the cytotoxic interaction of the dual combinations and mRNA expression of the efflux (*ABCB1* or *MDR1*, *ABCC1* or *MRP1*, *ABCC2* or *MRP2*, *ABCC3* or *MRP3*, *ABCC4* or *MRP4*, *ABCC11* or *MRP8*, and *ABCG2* or *BCRP*) and the uptake (*SLC28A1* or *hCNT1*, *SLC28A2* or *hCNT2*, *SLC28A3* or *hCNT3*, *SLC29A1* or *hENT1*, *SLC29A2* or *hENT2*, *SLC22A1* or *hOCT1*, and *SLC22A3* or *hOCT3*) transporters was investigated. Gemcitabine and cisplatin were selected as the interacting drugs in the study because their combination represents the current standard first-line chemotherapy regimen for advanced CCA. In addition, 5FU has been widely used in the treatment of biliary tract cancers. Importantly, the cytotoxic activity of these drugs is closely associated with cellular drug transport mechanisms, including nucleoside transporters such as *hENT1* and efflux transporters belonging to the ATP-binding cassette (ABC) family, which play critical roles in the development of chemoresistance.

## 2. Results

### 2.1. Cytotoxic Activity of Dual Combinations

Table 1 summarizes the cytotoxic activities of AT, BE, and the standard drugs GEM, Cis, and 5FU. The potencies of AT and BE activities across all cell lines were generally comparable to 5FU, with IC_50_ values ranging from 201.08 to 326.31 µM. All cell lines were most sensitive to GEM (IC_50_ 6.31–52.69 µM), followed by Cis (IC_50_ 18.90–62.06 µM) and 5FU (IC_50_ 226.26–1026.75 µM).

**Figure 2:**
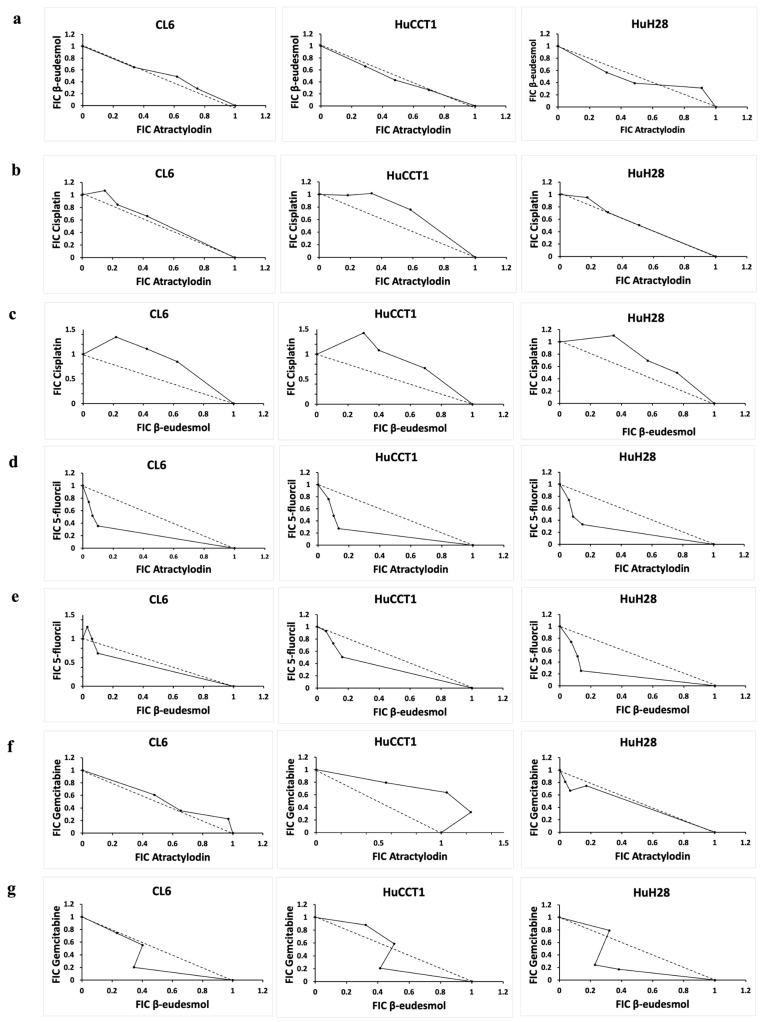
Isobolograms of the sum FIC values [median (range) of three independent experiments, triplicate each] of the dual combinations of the two compounds isolated from AL and types of interaction in CL6, HuCCT1, and HuH28 cell lines: (**a**) AT-BE combination (additive effect with sum FIC of 1.00 (0.786–1.286), 1.00 (0.761–1.172) and 1.00 (0.632–1.447), respectively), (**b**) AT-CIS combination (additive effect with sum FIC of 1.02 (0.921–1.253), antagonistic effect with sum FIC of 1.16 (1.000–1.489), and additive effect with sum FIC of 1.00 (0.956–1.242), respectively), (**c**) BE-CIS combination (antagonistic effect with sum FIC of 1.47 (1.000–1.622), 1.21 (1.000–1.820), and 1.22 (1.000–1.510), respectively), (**d**) AT-5-FU combination (synergistic effect with sum FIC of 0.77 (0.417–1.000), 0.83 (0.387–1.000), and 0.80 (0.401–1.000), respectively), (**e**) BE-5-FU combination (additive effect with sum FIC of 1.00 (0.679–1.292), 0.97 (0.480–1.131), and synergistic effect with sum FIC of 0.89 (0.377–1.007), respectively), (**f**) AT-GEM combination (additive effect with sum FIC of 1.00 (1.000–1.197), antagonistic effect with sum FIC of 1.35 (1.000–1.680), and additive effect with sum FIC of 0.92 (0.735–1.000), respectively), and (**g**) BE-GEM combination (additive effect with sum FIC of 0.97 (0.547–0.975), 1.00 (0.621–1.203), and 1.00 (0.470–1.112), respectively).

For dual combinations, the median (range) of sum FIC indexes indicative of the type of cytotoxic interaction suggested additive (AT-BE and BE-GEM for all cell lines; AT-Cis and AT-GEM for CL-6 and HuH28 cell lines; BE-5FU for CL-6 and HuCCT1 cell lines), synergistic (AT-5FU for all cell lines and BE-5FU for HuH28), and antagonistic (BE-Cis for all cell lines; AT-Cis and AT-GEM for HuCCT1) interactions (Table 2). The isobologram analyses of the FICs of the three dual combinations are shown in Figure 2. The dose–response curves showed that all three CCA cell lines synergized with AT-5-FU treatment (Supplementary Figures S5–S11).

### 2.2. Modulatory Effects of Dual Combinations on the Expression of Transporter Genes

**Figure 3:**
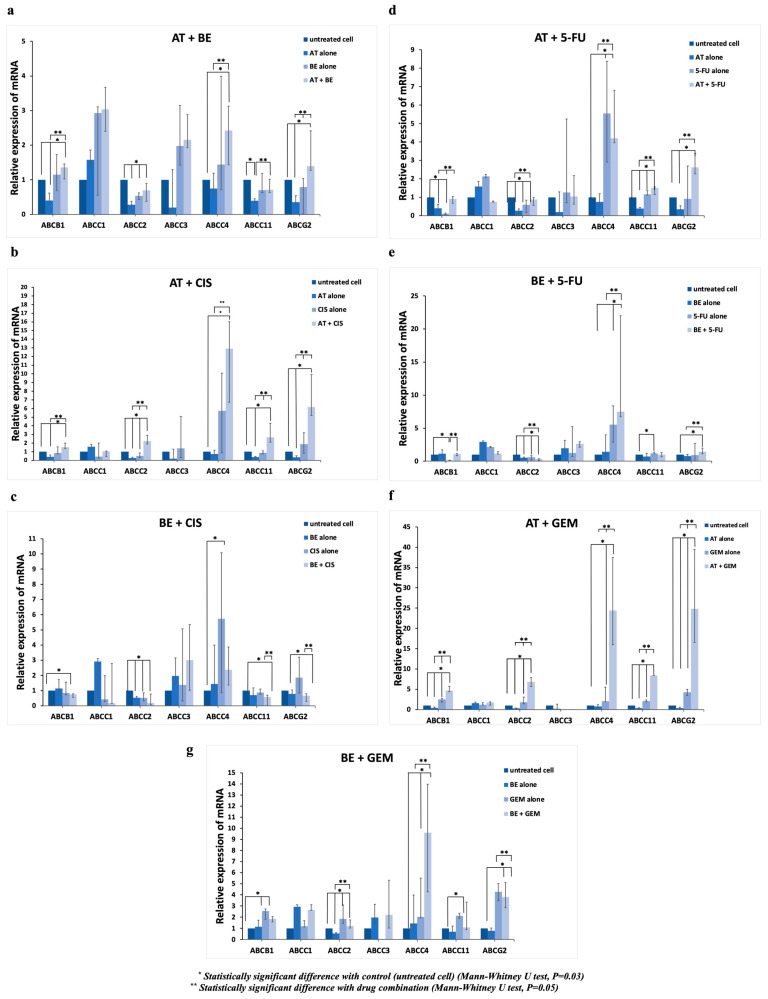
mRNA expression (RT-PCR analysis) of the efflux transporters in CL6 cell line following exposure to (**a**) AT-BE combination, (**b**) AT-Cis combination, (**c**) BE-Cis combination, (**d**) AT-5FU combination, (**e**) BE-5FU combination, (**f**) AT-GEM combination, and (**g**) BE-GEM combination. Each level of mRNA expression was normalized with GAPDH expression. Data are presented as median (range) values of three independent experiments (triplicate each). Statistical significance was determined with the Mann–Whitney U test (statistically significant difference with untreated control cells, a *, *p* = 0.03; statistically significant difference between single and combination treatment, **, *p* = 0.05).

**Figure 4:**
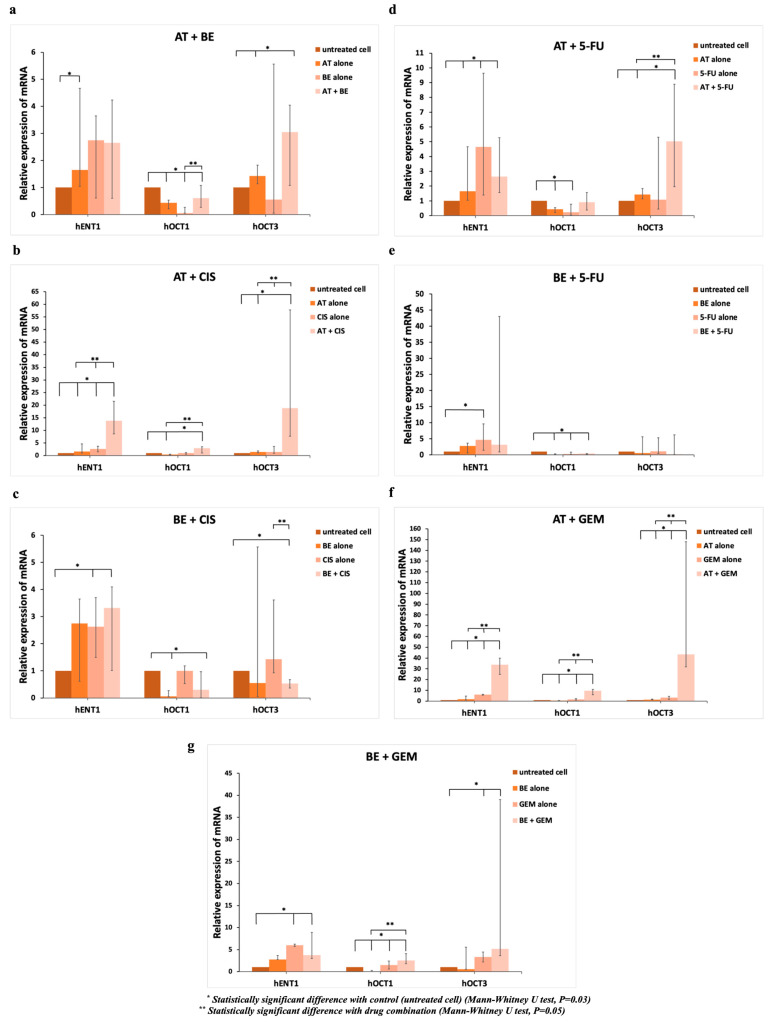
mRNA expression (RT-PCR analysis) of the influx transporters in CL-6 cell line following exposure to (**a**) AT-BE combination, (**b**) AT-Cis combination, (**c**) BE-CIS combination, (**d**) AT-5FU combination, (**e**) BE-5FU combination, (**f**) AT-GEM combination, and (**g**) BE-GEM combination. Each level of mRNA expression was normalized by GAPDH mRNA. Data are presented as median (range) values of three independent experiments (triplicate each). Statistical significance was determined with the Mann–Whitney U test (Statistically significant difference with untreated control cells, * *p* = 0.03, Statistically significant difference between single and combination treatment ** *p* = 0.05).

The mRNA expression profiles of the efflux transporters (*MDR1*, *MRP1*, *MRP2*, *MRP3*, *MRP4*, *MRP8*, and *BCRP*) and the reuptake transporters (*hCNT1*, *hCNT2*, *hCNT3*, *hENT1*, *hENT2*, *hOCT1*, and *hOCT3*) were investigated via RT-PCR analysis in the representative CCA cell line CL-6 after exposing cells to the test compounds (AT and BE) and the reference drugs (GEM, Cis, and 5FU) (Figure 3 and Figure 4 and Supplementary Tables S1 and S2).

#### 2.2.1. Efflux Transporters

AT treatment alone significantly decreased mRNA expression of *MDR1*, *MRP2*, *MRP8*, and *BCRP* by 0.39-, 0.28-, 0.39-, and 0.35-fold, respectively, compared with the untreated control. BE, Cis, and 5FU treatment alone significantly decreased *MRP2* expression by 0.53, 0.52, and 0.60-fold, respectively. 5FU alone significantly decreased *MDR1* expression by 0.105-fold. On the other hand, 5FU alone significantly increased *MRP4* and *MRP8* expression by 5.54 and 1.16-fold, respectively. GEM treatment alone also significantly increased the expression of *MDR1*, *MRP2*, *MRP4*, *MRP8*, and *BCRP* by 2.54-, 1.84-, 2.02-, 2.13-, and 4.27-fold, respectively.

Compared with each drug alone, the BE-Cis combination significantly decreased mRNA expression of *MRP8* (0.58-fold) and *BCRP* (0.66-fold), while the BE-5FU combination significantly decreased mRNA expression of *MRP2* (0.34-fold). On the other hand, the BE-5FU combination significantly increased the expression of *MDR1*, *MRP4*, and *MRP8* (1.06, 7.51, and 1.42-fold, respectively). Furthermore, the AT-Cis, AT-5FU and AT-GEM combinations significantly increased the expression of *MDR1* (1.51, 1.92, and 4.65-fold, respectively), *MRP2* (2.23, 1.87, and 6.81-fold, respectively), *MRP4* (12.90, 4.20, and 24.39-fold, respectively), *MRP8* (2.66, 1.50, and 8.40-fold, respectively) and *BCRP* (6.17, 2.62, and 24.82-fold, respectively). In addition, the AT-BE combination significantly increased *MRP4*, *MRP8*, and *BCRP* expression (2.43, 1.72, and 1.39-fold, respectively). The BE-GEM combination significantly increased *MRP2*, *MRP4*, and *BCRP* expression (1.20, 9.60, and 3.81-fold, respectively).

#### 2.2.2. Reuptake Transporters

The *hCNT1*, *hCNT2*, *hCNT3*, and *hENT2* genes were not detected in the CL-6 cell line, while other transporter genes were differentially expressed. AT, BE, and 5FU alone significantly decreased *hOCT1* expression by 0.44, 0.06, and 0.23-fold, respectively, compared with the control. In contrast, AT, Cis, 5FU, and GEM alone significantly increased *hENT1* expression by 1.65-, 2.63-, 4.64-, and 5.98-fold, respectively, compared with the control. GEM alone significantly increased *hOCT1* and *hOCT3* expression by 1.48 and 3.34-fold, respectively. AT alone significantly increased *hOCT3* expression by 1.42-fold.

Compared with each drug alone, AT-BE and BE-GEM combinations significantly increased mRNA expression of *hOCT1* (1.60 and 2.55-fold, respectively). AT-Cis and AT-GEM combinations significantly increased the expression of *hENT1* (13.87 and 33.83-fold, respectively), *hOCT1* (2.90 and 9.70-fold, respectively), and *hOCT3* (18.84 and 43.45-fold, respectively). In addition, the AT-5FU combination significantly increased the expression of *hOCT3* (5.03-fold). On the other hand, the BE-Cis combination significantly decreased mRNA expression of *hOCT3* (0.54-fold).

## 3. Discussion

In this study, the cytotoxic interactions between the two major bioactive compounds of AL (β-eudesmol and atractylodin; BE and AT) and conventional chemotherapeutic drugs were investigated in the three human CCA cell lines CL-6, HuCCT1, and HuH28. The possible link between the cytotoxic activities of standard medications when combined with either compound and the modulation of mRNA expression of efflux and reuptake transporter genes was investigated to explore the contribution of transporter genes to improving the cytotoxic activities of BE and AL with chemotherapeutic drugs. As the major bioactive constituents of AL rhizome, atractylodin (14% of dry weight) and β-eudesmol (6% of dry weight) were selected for the study. Both compounds significantly inhibited CCA cell growth, survival, migration, cell cycle arrest, and apoptosis. β-eudesmol and atractylodin inhibited CCA cell growth with comparable potency to 5FU (IC_50_ 226.26–1026.75 vs. 201.08–326.31 µM) [29]. All cell lines were obtained from patients with advanced-stage intrahepatic CCA: CL-6 (a Thai patient) and HuCCT1 and HuH28 (Japanese patients). Previously, we demonstrated that β-eudesmol induced apoptosis in CL-6 cells by 37.84%, but not in normal cells (OUMS-36T-1F) [30]. However, apoptosis induction by atractylodin was observed in both cells (27.21% and 31.74% for CL-6 and OUMS-36T-1F cells, respectively). Cell cycle arrest occurred at the G1 phase, and potent induction of apoptosis occurred at 48 h of exposure to both compounds. Furthermore, β-eudesmol treatment suppressed the colony-forming and wound-healing abilities of CL-6 cells in a concentration-dependent manner. Western blot analysis indicated that atractylodin and β-eudesmol treatment suppressed heme oxygenase-1 (HO-1) protein expression. HO-1 expression is essential for maintaining the cytoprotective activity of CL-6 cells. The downregulation of STAT1/3 phosphorylation and NF-κB (p65 and p50) protein expression by both compounds was concentration-dependent [30]. The anti-CCA activity of β-eudesmol was confirmed in a CCA-xenografted nude mouse model [36]. At a high dose (5000 mg/kg body weight), it potently inhibited tumour growth and prolonged survival time compared with 5FU and the untreated control.

The CL-6 cell line was selected for the mRNA expression analysis of transporter genes due to its consistent responsiveness to β-eudesmol and atractylodin [29,30,31]. This cell line was derived from a Thai patient with advanced-stage intrahepatic CCA, the primary risk factor for which is the consumption of improperly cooked fish containing metacercariae of the liver fluke *O. viverini*. Interestingly, the results of the present study showed that the AT-5FU and BE-5FU combinations produced synergistic cytotoxic effects in at least one of the three CCA cell lines (sum FIC: 0.417–1.000, 0.387–1.000, and 0.401–1.000) for CL-6, HuCCT1, and HuH28, respectively. The combinations of atractylodin with β-eudesmol (AT-BE), cisplatin (AT-Cis), or gemcitabine (AT-GEM) resulted in additive cytotoxic effects at least in the CL-6 cell line (sum FIC: 0.785–1.286). The combinations of BE with GEM (BE-GEM) or 5FU (BE-5FU) also resulted in additive cytotoxic effects, at least in CL-6 cells (sum FIC: 0.547–1.292). Antagonistic cytotoxic effects, on the other hand, were observed with the combinations of atractylodin with cisplatin (AT-Cis) or gemcitabine (AT-GEM) and β-eudesmol with cisplatin (BE-Cis) (sum FIC: 1.000–1.820). The synergistic interactions observed with the combination of atractylodin with 5FU (AT-5FU) and β-eudesmol with 5FU (BE-5FU) were well correlated with the significant upregulation of the expression of the reuptake transporters *hENT1* (2.64-fold vs. untreated control) and *hOCT3* (5.03-fold vs. each drug alone), as well as the significant downregulation of the expression of the efflux transporters *MRP2* (0.33-fold vs. each drug alone). While in vitro Selectivity Indices on non-cancerous cell lines (e.g., MMNK-1 or fibroblasts) were not generated in this specific panel, the established clinical safety data strongly support the safe application of these bioactive compounds in humans. Altogether, this led to the accumulation of AT and BE in the CCA cells, thereby increasing cell-killing effects. Although other transporters (reuptake and efflux) modulated the transport of AT, BE, and 5FU in opposite directions, *hENT*, *hOCT1*, and *hOCT3* are likely to be the main modulators that are involved in the increase in the chemosensitizing effects of 5FU by atractylodin and β-eudesmol. β-eudesmol was also shown to enhance the cytotoxicity of 5FU and doxorubicin by increasing apoptosis in CCA KKU-100 cells [31]. The effect correlated with the suppressive effect of β-eudesmol on NQO1 expression and enzyme activity, as well as its inhibitory effect on cell migration (through induction of cell apoptosis and activation of caspase 3/7). Atractylodin was also shown to downregulate the expression of other efflux transporters, *ABCA12* and *ABCG8*, in CL-6 cells [32]. The antagonistic effects observed with the combinations of atractylodin with cisplatin (AT-Cis) or gemcitabine (AT-GEM) and β-eudesmol with cisplatin (BE-Cis) were in parallel with the upregulation of the efflux transporters *MDR1* (1.38–5.72-fold), *MRP2* (1.89–7.90-fold), *MRP4* (6.71–37.46-fold), MRP8 (2.12–8.49-fold), and *BCRP* (5.19–39.47-fold), together with the downregulation of the reuptake transporters *hOCT1* (0.306-fold) and *hOCT3* (0.539-fold). These led to decreased accumulation of atractylodin, β-eudesmol, cisplatin, and/or gemcitabine in CCA cells. 5FU and gemcitabine are taken up into cells through nucleoside transporters, ENTs and CNTs [18]. Downregulation of *hENT1* was found in gemcitabine-resistant CCA cell lines KKU-M139/GEM and KKU-M214/GEM) [21]. Both cell lines showed upregulation of multidrug resistance protein 1 (*MRP1*/*ABCC1*), leading to increased drug efflux. While our current analysis focused on mRNA expression levels of a range of efflux and reuptake transporters, this does not definitively establish a causal relationship between these transporters and the observed drug interactions. Identification of the specific transporters responsible for the synergistic effects of AT-5FU, as well as the potential additive or antagonistic effects seen with other combinations. To address this, we can include additional experimental techniques, e.g., Western blotting and immunohistochemistry (IHC), to assess transporter expression at the protein level and verify the correlation between mRNA and protein levels, thereby providing a more comprehensive understanding of transporter activity in relation to drug effects. The incorporation of additional experiments, including transporter knockdown and overexpression models, will confirm the role of key transporters in mediating the cytotoxicity of the drug combination interactions. Furthermore, incorporating several additional assays, including apoptosis, cell cycle, and migration and invasion assays, will allow evaluation of the effects of drug combinations on cell death, proliferation, and metastatic potential across different cell lines.

The efflux transporter adenosine triphosphate-binding cassette (ABC) superfamily plays an essential role in determining the intracellular concentrations of drugs [22]. MRP4, MRP5, and MRP8 have been shown to efflux cyclic nucleotides and nucleoside analogs, thereby conferring drug resistance [37]. Non-specific induction of the expression of ABC transporters, including *ABCB1* (*MDR1*), *ABCG2* (*BCRP*), *ABCC10* (*MRP7*), and *ABCC11* (*MRP8*), was shown to reduce the sensitivity of CCA cells to gemcitabine [38]. The contribution of the efflux transporters *MDR1* and *BCRP* to the expression profile of gemcitabine resistance and tumour relapse has previously been reported [39]. P-gp (*ABCB1*), encoded by the multidrug resistance 1 (*MDR1*) gene, is expressed in liver tissues and has been associated with 5FU resistance [40]. The present study showed that atractylodin alone significantly downregulated the mRNA expression of *MDR1*, *MRP2*, *MRP8*, and *BCRP* by about 0.39-, 0.28-, 0.39-, and 0.35-fold, respectively, compared with the untreated control. However, upregulation of *MDR1*, *MRP2*, *MRP4*, *MRP8*, and *BCRP* expression was observed when atractylodin was combined with standard drugs (Figure 3b,d,f). Cisplatin is a platinum derivative that is taken into the cells by SLC transporters, including OCT [41]. A decrease in mRNA expression and/or activity of the reuptake transporters *OCT3* (*SLC22A3*) and *OCT1* (*SLC22A1*) has been reported in CCA [20]. In addition, low *SLC22A1* expression was significantly associated with poor survival in CCA patients [22]. Cisplatin-resistant cells showed a stable reduction in cisplatin accumulation and a downregulation of *OCT3*. In contrast, *OCT3* overexpression reverses the resistance [39]. Our results showed significant upregulation of *hENT1* (13.87-fold), *hOCT1* (2.90-fold), and *hOCT3* (18.84-fold) when the cells were exposed to the combination of atractylodin and cisplatin (AT-Cis) compared with each drug alone. Atractylodin treatment alone significantly upregulated *hOCT3* expression by 1.42-fold. In contrast, significant downregulation of *hOCT1* expression was observed when cells were exposed to atractylodin, β-eudesmol, or 5FU alone (0.44-, 0.057-, or 0.23-fold, respectively). It was noted that the expression of *hCNT*, *hCNT2*, *hCNT3*, and *hENT2* in CL-6 cells was absent. The antagonistic effects observed with AT and BE combinations with cisplatin and gemcitabine can be, at least in part, attributed to a combination of transporter modulation (upregulation of efflux transporters and downregulation of reuptake transporters), changes in cellular signaling pathways (such as STAT1/3 and NF-κB) [30], potential competitive interactions between the drugs at the transporter level, and alterations in DNA repair mechanisms. These mechanisms collectively reduce intracellular accumulation of cisplatin and gemcitabine, thereby decreasing their cytotoxicity. Understanding these interactions at the molecular level is crucial for optimizing combination therapies and overcoming resistance mechanisms in CCA treatment.

Several studies have demonstrated that polymeric nanoparticles encapsulating curcumin can inhibit the efflux of drugs mediated by P-glycoprotein (P-gp), multidrug resistance-associated protein 1 (*MRP1*), and breast cancer resistance protein (*BCRP*), thereby restoring drug sensitivity in multidrug-resistant (MDR) cancer cells [42,43]. Tetrandrine (TET), a bisbenzylisoquinoline alkaloid derived from the root of *Stephania tetrandra* (Fenfangji), has been shown to reverse MDR in vitro by affecting P-gp-mediated drug efflux in cancer cells [44]. Similarly, piperine, a compound found in black pepper (*Piper nigrum*) and long pepper (*Piper longum*), enhances the intracellular accumulation of P-gp substrates like rhodamine and calcein, while inhibiting their efflux in MDR cell lines [45]. Prolonged exposure to piperine also suppresses the transcription of P-gp genes. Kaempferol and myricetin have been reported to downregulate P-gp and *MRP1* expression in promyelocytic leukemia and osteosarcoma cells, respectively [46]. Both kaempferol and myricetin exhibit potent inhibition of MRP1 activity. Additionally, quercetin has been shown to reduce *MRP1* expression in gastric adenocarcinoma cells, thereby reversing the drug-resistant phenotype. Similar modulatory effects on multidrug resistance transporters have also been reported for several other polyphenolic compounds, suggesting that natural phytochemicals may help overcome chemotherapy resistance by regulating ABC transporter expression [47]. Quercetin has also been found to enhance the efficacy of cisplatin and increase the sensitivity of breast cancer cell lines to doxorubicin [48].

In the present study, the combination of atractylodin or β-eudesmol with cisplatin and gemcitabine showed a tendency toward antagonism in cytotoxicity in all CCA cell lines (CL-6, HuCCT1, and HuH28). β-eudesmol and atractylodin, as purified compounds or major components of the AL extract, are expected to provide beneficial therapeutic effects on CCA when used as an adjunct to standard chemotherapeutic drugs, particularly 5FU. Combination therapy with cisplatin and gemcitabine may result in antagonistic cytotoxic activity. The mechanism of the synergistic activity may, in part, be due to upregulation of the reuptake transporter (*hOCT3*) and downregulation of the efflux transporter (*ABCC2*) in CCA cells. Future pharmacokinetic studies, potentially using molecular docking simulations and enzyme kinetics assays, are necessary to distinguish between these transcriptional effects and direct competitive binding interactions.

We investigated modulation of drug transporter genes as a key mechanism underlying the synergistic or antagonistic cytotoxic effects observed when β-eudesmol and atractylodin were combined with chemotherapeutic agents in CCA cells. While drug influx and efflux regulation play a significant role, the anticancer potential of BE and AT likely involves additional cellular mechanisms. Evidence from our previous studies suggests that both compounds exert multi-targeted effects. They inhibit critical signalling pathways such as Notch1, PI3K/AKT/mTOR, and MAPK, which are associated with cancer cell survival, angiogenesis, and therapy resistance [49,50,51]. Future studies will focus on validating downstream effects on cell cycle distribution and apoptotic signaling cascades (e.g., caspase activation) to substantiate these findings. Furthermore, BE and AT activate caspase-dependent apoptosis (e.g., Bax, cleaved caspase-3) and induce cell cycle arrest at G0/G1 or G2/M phases via modulation of cyclin-CDK complexes [52,53]. Both compounds also suppress epithelial–mesenchymal transition (EMT) by downregulating vimentin and N-cadherin, while upregulating E-cadherin, thereby reducing metastatic potential [49]. In addition to their cytotoxic effects, BE and AT modulate pro-inflammatory cytokines and mediators, such as TNF-α, IL-6, and COX-2, suggesting roles in immune modulation and regulation of the tumour microenvironment [54,55]. While these findings support the therapeutic promise of BE and AT in CCA, several limitations remain. These include the lack of definitive causal validation of transporter activity at the protein level, limited in vivo exploration, and the need for broader cell-line validation beyond CL-6. Furthermore, while mRNA expression is a strong indicator, it does not guarantee a linear relationship with protein abundance, and future protein-level validation is planned to confirm these mechanistic insights. Functional validation using quantitative protein assays (Western blot/immunohistochemistry) and/or specific transporter inhibitors or siRNA knockdown models is the next immediate step in our research pipeline to confirm the causal roles of *hENT1* and *MRPs*. The relationship between cytotoxic interactions, transporter expression, and plasma drug concentrations warrants investigation in animal models and human studies. Future work should validate these mechanisms in vivo, explore clinical applications, and assess the relevance of transporter profiles in patient-derived samples to better understand how BE and AT may enhance the efficacy of conventional chemotherapy in CCA.

This study highlights the significant role of drug transporter modulation by atractylodin (AT) and β-eudesmol (BE) in shaping their synergistic or antagonistic cytotoxic interactions with conventional chemotherapeutic agents in CA cells. Notably, the upregulation of *hENT1* and *hOCT3*, along with the downregulation of *MRP2*, in the presence of AT and BE combined with 5FU suggests that AT and BE may modulate the expression of SLC transporters, thereby potentially enhancing the cellular uptake of chemotherapeutic agents such as 5FU. We also acknowledge that whether AT and BE are direct substrates of *hENT1* or *hOCT3* remains unclear and requires further investigation. Conversely, combinations such as AT-Cis and BE-Cis, which induced efflux transporter upregulation, may lead to reduced intracellular drug levels and diminished efficacy. While these findings support the potential of AT and BE as chemosensitizing agents, several challenges must be addressed before clinical application. As phytochemicals derived from *A. lancea*, AT and BE are generally regarded as less toxic than synthetic drugs, potentially offering a safer adjunct option in CCA treatment, particularly for elderly or chemotherapy-intolerant patients. However, in vitro synergism does not always reflect in vivo outcomes due to the complexities of the tumor microenvironment, host immune responses, and pharmacokinetic variables. To bridge this translational gap, further studies are essential. Comprehensive preclinical evaluations should focus on safety, pharmacokinetics, organ-specific toxicity, and potential herb-drug interactions. This is especially important in vulnerable populations such as elderly patients with advanced-stage intrahepatic cholangiocarcinoma (iCCA), who often decline or cannot tolerate standard regimens. A stepwise clinical trial pathway is proposed, beginning with phase I studies to determine optimal dosing, tolerability, and interaction profiles with chemotherapeutics. Phase II trials should follow to assess preliminary efficacy, particularly in patients refractory to or unsuitable for standard chemotherapy. Given the observed synergism with low-dose 5FU, AT and BE may hold therapeutic promise in low-resource settings such as Northeastern Thailand, where effective, tolerable alternatives to conventional chemotherapy are urgently needed.

## 4. Materials and Methods

### 4.1. Cell Lines

The human CCA cell lines CL-6, HuCCT1, and HuH28 were used in the study. The CL-6 cell, originally isolated from the tumour tissue of a CCA patient at Siriraj Hospital (Mahidol University, Bangkok, Thailand), was kindly provided by Associate Professor Adisak Wongkajornslip. The HuCCT1 and HuH28 cells were purchased from the Japanese Collection of Research Bioresources (JCRB) Cell Bank, Ibaraki, Japan.

### 4.2. Chemicals and Reagents

Atractylodin (AT), β-eudesmol (BE), and 5-fluorouracil (5FU, ≥98% (HPLC) were purchased from Wako Pure Chemical Industries (Osaka, Japan). Gemcitabine (GEM, ≥98% (HPLC)) and cisplatin (Cis, ≥98% (HPLC)) were obtained from Sigma-Aldrich Company (St. Louis, MO, USA). 3-[4,5-dimethylthiazole-2-yl]-2,5-diphenyltetrazolium bromide (MTT) was purchased from Life Technologies (Carlsbad, CA, USA). The cell culture medium Roswell Park Memorial Institute (RPMI-1640), fetal bovine serum (FBS), trypsin-EDTA (0.25%), Antibiotic-Antimycotic (100×) and dimethyl sulfoxide (DMSO) were purchased from Gibco BRL Life Technologies (Grand Island, NY, USA). Trizol reagent was purchased from Life Technologies (Carlsbad, CA, USA). HPLC-grade ethanol and isopropanol were purchased from Thermo Fisher Scientific (Newington, NH, USA).

### 4.3. Preparation of Test Materials and Reference Drugs

AT, BE, and 5FU were initially dissolved in 50% ethanol. GEM and Cis were dissolved in distilled water. The concentrated stock solution of each test compound/drug was prepared by adding the known weight to a calculated volume of 50% ethanol or distilled water, and the working solution was prepared freshly before use.

### 4.4. Cell Culture

CL-6, HuCCT1, and HuH28 were cultured in complete RPMI media supplemented with 10% heated FBS and 1% Antibiotic-Antimycotic solution. All cells were maintained under 5% CO2 at 37 °C and 95% humidity (HERA CELL 150i, Thermo Scientific, Waltham, MA, USA). The cells were subcultured twice a week; 0.25% Trypsin-EDTA was added, and the cells were separated through centrifugation at 1500× *g* for 5 min. The study protocol was approved by the Institutional Biosafety Committee of Thammasat University (TU-IBC 105/2560, dated 12 January 2021). The CCA cell lines were maintained as described in Supplementary Figure S1.

### 4.5. Analysis of the Cytotoxic Activity of Dual Combinations

CL-6, HuCCT1, and HuH28 were seeded (0.8 × 10^4^ cells/well) onto a 96-well plate and pre-incubated at 37 °C for 24 h. The cytotoxic interaction of each dual combination, i.e., AT-BE, AT-5-FU, AT-Cis, AT-GEM, BE-5FU, BE-Cis and BE-GEM was determined at the concentration ratios 10:0, 7:3, 5:5, 3:7, and 0:10 (serial dilution for each combination pair). The highest concentrations of each compound/drug used were 200 µg/mL (AT and BE), 1000 µg/mL (5FU), 100 µg/mL (Cis), 10 µg/mL (GEM) for CL-6 and HuCCT1 cell lines, and 100 µg/mL (GEM) for HuH28 cell line. The culture was incubated at 37 °C for 48 h, and the MTT reagent (20 µL) was added to each well, and the culture was further incubated for four hours. The cell suspension was carefully removed, and DMSO was added. The absorbance was measured at 590 nm within 15 min. The experiment was repeated at least three times (triplicate each). The concentrations that inhibit cell growth by 50% (IC_50_) and 25% (IC_25_) of each compound (from the starting ratios of 10:0 and 0:10) were estimated using Calcusyn™ v1.1. (Biosoft, Cambridge, UK). Data were fitted to a dose–response curve using Calcusyn™ (Biosoft, Cambridge, UK) to obtain the concentrations that inhibit cell growth by 50% (IC_50_) and 25% (IC_25_) of each compound (from the starting ratios of 10:0 and 0:10). Cell survival for all experiments was expressed as of viable cells relative to that in untreated cells (defined as 1.0). The Fractional Inhibitory Concentration (FIC) calculation evaluates the interaction between two drugs in combination therapy. It provides a measure of the effect of the combination compared to each drug used alone. Each combination pair’s FIC (representing combination scores) and the sum FIC of five distinctive ratios were calculated as the ratio of IC_50_ of the combination and that of each compound alone. The isobologram of each combination interaction was generated from the average sum FIC index, which was estimated from the sum of the IC_50_ of each combination pair divided by the IC_50_ of every single compound. The sum FIC of <1, =1, and >1 indicates synergistic, additive, and antagonistic interactions, respectively.

### 4.6. RNA Extraction and RT-PCR Analysis

The CL-6 cell was seeded (1.5 × 10^6^ cells/flask) on a cell culture flask and pre-incubated at 37 °C for 24 h. The cell was incubated with the corresponding IC_25_ concentration of each dual combination (AT-BE, AT-5FU, AT-Cis, AT-GEM, BE-5FU, BE-Cis, and BE-GEM) for an additional 48 h. Total RNA was extracted using TRIzol^®^ Reagent (Life Technologies, Carlsbad, CA, USA) according to the manufacturer’s recommendation. Chloroform and isopropanol were used to purify and precipitate RNA, respectively. The obtained RNA was washed with 75% ethanol, dried, and dissolved in diethylpyrocarbonate (DEPC)-treated water. RNA and DNA concentrations were measured using NanoDrop spectroscopy (Thermo Fischer Scientific, Waltham, MA, USA). Single-stranded cDNA was prepared from total RNA (1 μg) using SuperScript™ III Reverse Transcriptase cDNA construction kit (Life Technologies, Carlsbad, CA, USA) following the manufacturer’s instructions. A no-template negative control and glyceraldehyde-3-phosphate dehydrogenase (GAPDH, a normalization reference gene) were included in the experiment. The PCR primers and the expected PCR product sizes are shown in Table 3. Supplementary Figures S1–S4 display the PCR products of efflux and reuptake transporter genes analyzed by gel electrophoresis. Real-time PCR (RT-PCR) was performed using iTaq™ Universal SYBR^®^ Green Supermix (Bio-Rad Laboratories, Hercules, CA, USA) according to the manufacturer’s instructions. Reaction conditions for the amplification of ATP-binding cassette transporters (*MDR1*, *MRP1*, and *MRP2*) were initiated by denaturation at 95 °C for 10 min, followed by 40 repeated cycles of 95 °C for 15 s and 60 °C for 1 min [56]. In addition, the amplification of *MRP3*, *MRP4*, *MRP8*, and *BCRP* was initiated by pre-incubation at 95 °C for 5 min, followed by 40 repeated cycles of 95 °C for 10 s, 55 °C for 10 s, and 72 °C for 3 s [22]. Reaction conditions for the amplification of human nucleoside transporters (*hCNT1*, *hCNT2*, *hCNT3*, *hENT1*, and *hENT2*) were: 15 min at 95 °C, 40 cycles of 1 min at 94 °C, 30 s at 65 °C [57]. The amplification of human organic cation transporters (*hOCT1* and *hOCT3*) was initiated by pre-incubation at 95 °C for 3 min, followed by 30 s at 94 °C, 1 min at 60 °C, and 1 min at 72 °C for 40 cycles [58]. Samples were run on a CFX96 RT-PCR system (Bio-Rad, CA, USA). Relative quantification of gene expression was assessed using the 2-ΔΔCT method, where ΔΔCT = (CT_target gene_ − CT_reference gene_) treatment − CT_target gene_ − CT_reference gene_) control, and CT represents the cycle threshold for a given gene. The experiment was repeated in three separate experiments (triplicate each), and data are presented as median (range) values.

### 4.7. Statistical Analysis

The nonparametric analysis was applied to data that did not conform to normality, and quantitative data were summarized as median (range) values. The Mann–Whitney U test was used to compare changes in mRNA expression of efflux and reuptake transport proteins in CL-6 cells following AL exposure versus untreated controls. between two independent quantitative variables. The statistical significance level was set at α = 0.05 for all tests (SPSS for Windows, version 12; IBM, New York, NY, USA).

## 5. Conclusions

Our findings demonstrate that atractylodin and beta-eudesmol significantly enhance the cytotoxicity of 5FU in CCA cell lines. The observed synergistic interactions are closely associated with the transcriptional modulation of key drug transporters (upregulation of *hENT1*/*hOCT3* and downregulation of *ABCC2*). These results suggest a putative mechanism wherein these bioactive compounds may facilitate drug accumulation, providing a rationale for their use as chemosensitizers. Future studies focusing on protein quantification and functional transport assays are required to confirm this mechanism definitively.

## Figures and Tables

**Table 1:** Cytotoxic activities (presented as IC_50_ and IC_25_ values) of the two bioactive compounds of AL, atractylodin (AT) and β-eudesmol (BE), and conventional chemotherapeutic drugs 5-fluorouracil (5FU), cisplatin (Cis), and gemcitabine (GEM) against CCA cell lines (CL-6, HuCCT1, and HuH28) using MTT assay. Data are presented as median (range) from independent experiments, each in triplicate.

Cytotoxic Activity				
Cell Lines	CL6		HuCCT1	HuH28
Compounds	IC_50_ (µM)	IC_25_ (µM)	IC_50_ (µM)	IC_50_ (µM)
AT	197.29 (201.08–241.91)	131.43 (118.98–157.72)	210.89 (198.61–234.83)	276.59 (247.39–326.31)
BE	198.36 (196.70–203.26)	124.93 (53.92–164.37)	180.24 (169.94–223.37)	202.14 (158.88–202.46)
5FU	250.46 (226.26–540.17)	11.15 (9.84–43.90)	613.05 (588.14–666.03)	958.41 (533.56–1026.75)
Cis	25.39 (18.90–31.08)	8.22 (4.66–10.18)	31.18 (26.46–36.47)	59.00 (48.92–62.06)
GEM	8.91 (7.98–14.05)	4.17 (0.0009–4.54)	7.07 (6.57–7.71)	40.15 (6.31–52.69)

**Table 2:** The sum FIC values (median (range)) of the dual combinations of the two bioactive compounds from AL, atractylodin (AT) and β-eudesmol (BE), with conventional chemotherapeutic drugs 5-fluorouracil (5FU), cisplatin (Cis), and gemcitabine (GEM) against CCA cell lines (CL-6, HuCCT1, and HuH28) using MTT assay. Data are presented as median (range) from independent experiments, each performed in triplicate.

Sum FIC Median (Range)			
Dual Combinations	CL6	HuCCT1	HuH28
AT-BE	1.00 (0.786–1.286)	1.00 (0.761–1.172)	1.00 (0.632–1.447)
AT-Cis	1.02 (0.921–1.253)	1.16 (1.000–1.489)	1.00 (0.956–1.242)
BE-Cis	1.47 (1.000–1.622)	1.21 (1.000–1.820)	1.22 (1.000–1.510)
AT-5FU	0.77 (0.417–1.000)	0.83 (0.387–1.000)	0.80 (0.401–1.000)
BE-5FU	1.00 (0.679–1.292)	0.97 (0.480–1.131)	0.89 (0.377–1.007)
AT-GEM	1.00 (1.000–1.197)	1.35 (1.000–1.680)	0.92 (0.735–1.000)
BE-GEM	0.97 (0.547–0.975)	1.00 (0.621–1.203)	1.00 (0.470–1.112)

**Table 3:** Primers used for RT-PCR.

Gene	Primers	Primer Sequence (5′–3′)	Product Sizes (bp)	Ref.
*ABCB1* *(MDR1)*	Forward	GTCTTTGGTGCCATGGCCGT	206	[1]
	Reverse	ATGTCCGGTCGGGTGGGATA		
*ABCC1* *(MRP1)*	Forward	CTGACAAGCTAGACCATGAATGT	262	
	Reverse	CCTTTGTCCAAGACGATCACCC		
*ABCC2* *(MRP2)*	Forward	GCCAGATTGGCCCAGCAAA	202	
	Reverse	AATCTGACCACCGGCAGCCT		
*ABCC3* *(MRP3)*	Forward	CCTGCTCTCCTTCATCAATC	156	[2]
	Reverse	ATGTAGTGGTAATAGTGTTGTAAG		
*ABCC4* *(MRP4)*	Forward	TACAAGTGGTTGGTGTGGTCTCTG	143	
	Reverse	TGTAGATTCCAGGCGCTTCACA		
*ABCC11* *(MRP8)*	Forward	TAGCTGAAAGAATTGGCAGGAACT	242	
	Reverse	TCATGGTTCTCAAGGCAGCATC		
*ABCG2* *(BCRP)*	Forward	CACCTTATTGGCCTCAGGAA	206	
	Reverse	CCTGCTTGGAAGGCTCTATG		
*SLC28A1* *(hCNT1)*	Forward	CATTACTGATCCGGCCCTACTT	75	[3]
	Reverse	TGGCGTAACCTCCGGTCAT		
*SLC28A2* *(hCNT2)*	Forward	CTTGTGCTCTCGCCTCATCA	75	
	Reverse	TTACCCCCTCCTCACTCTTGAA		
*SLC28A3* *(hCNT3)*	Forward	ATTGCTGGAAGCGTGCTAGGT	90	
	Reverse	TGACGCAGGTGCTGACATAAC		
*SLC29A1* *(hENT1)*	Forward	TCTCCAACTCTCAGCCCACCAA	151	
	Reverse	CCTGCGATGCTGGACTTGACCT		
*SLC29A2* *(hENT2)*	Forward	ATGAGAACGGGATTCCCAGTAG	81	
	Reverse	GCTCTGATTCCGGCTCCTT		
*SLC22A1* *(hOCT1)*	Forward	GTGTGTAGACCCCCTGGCTA	363	[4]
	Reverse	GTGTAGCCAGCCATCCAGTT		
*SLC22A3* *(hOCT3)*	Forward	ATCGTCAGCGAGTTTGACCT	324	
	Reverse	TTGAATCACGATTCCCACAA		
*GAPDH*	Forward	TGAAGGTCGGAGTCAACGGATTTG	628	[5]
	Reverse	GCGCCAGTAGAGGCAGGGATGATG		

## Data Availability

All data generated or analyzed during this study are included in the published article.

## Supplementary Materials

The following supporting information can be downloaded at: https://www.mdpi.com/article/10.3390/molecules31071124/s1, Figure S1. Agarose gel showing the PCR products obtained from CL-6 cell lines with primer targeting housekeeping gene and efflux transporter genes. Figure S2. Agarose gel showing the PCR products obtained from CL-6 cell lines with primer targeting housekeeping and reuptake transporter genes. Figure S3. Agarose gel showing the PCR products obtained from Caco2 cell lines (as a positive control) with primer targeting housekeeping and efflux transporter genes. Figure S4. Agarose gel showing the PCR products obtained from Caco2 cell lines (as a positive control) with primer targeting housekeeping and reuptake transporter genes. Figure S5. Dose-response curves in CL-6 (a), HuCCT1 (b), and HuH28 (c) cell lines combining atractylodin (AT) and β-eudesmol (BE) at ratios of 7:3, 5:5, and 3:7 for 48 h. Figure S6. Dose-response curves in CL-6 (a), HuCCT1 (b), and HuH28 (c) cell lines combining atractylodin (AT) and cisplatin (CIS) at ratios of 7:3, 5:5, and 3:7 for 48 h. Figure S7. Dose-response curves in CL-6 (a), HuCCT1 (b), and HuH28 (c) cell lines combining β-eudesmol (BE) and cisplatin (CIS) at ratios of 7:3, 5:5, and 3:7 for 48 h. Figure S8. Dose-response curves in CL-6 (a), HuCCT1 (b), and HuH28 (c) cell lines combining atractylodin (AT) and 5-fluorouracil (5-FU) at ratios of 7:3, 5:5, and 3:7 for 48 h. Figure S9. Dose-response curves in CL-6 (a), HuCCT1 (b), and HuH28 (c) cell lines combining β-eudesmol (BE) and 5-fluorouracil (5-FU) at ratios of 7:3, 5:5, and 3:7 for 48 h. Figure S10. Dose-response curves in CL-6 (a), HuCCT1 (b), and HuH28 (c) cell lines combining atractylodin (AT) and gemcitabine (GEM) at ratios of 7:3, 5:5, and 3:7 for 48 h. Figure S11. Dose-response curves in CL-6 (a), HuCCT1 (b), and HuH28 (c) cell lines combining β-eudesmol (BE) and gemcitabine (GEM) at ratios of 7:3, 5:5, and 3:7 for 48 h. Table S1. Relative mRNA expression, compared to *GAPDH* expression, of the efflux transporters in CL6 cell line following exposure to atractylodin (AT) and β-eudesmol (BE) alongside standard chemotherapies for CL-6, including 5-FU, cisplatin, and gemcitabine. Table S2. Relative mRNA expression, compared to GAPDH expression, of the uptake transporters in CL6 cell line following exposure to atractylodin (AT) and β-eudesmol (BE) alongside standard chemotherapies for CL-6, including 5-FU, cisplatin, and gemcitabine.

## Abbreviations

The following abbreviations are used in this manuscript:

AL	*Atractylodes lancea*
5FU	5-fluorouracil
ABC	Adenosine triphosphate-binding cassette
ABCA12	ATP-binding cassette sub-family A member 12
ABCG8	ATP-binding cassette sub-family G member 8
AT	Atractylodin
BCRP	Breast cancer resistance protein
BE	β-eudesmol
CCA	Cholangiocarcinoma
Cis	Cisplatin
CNTs	Concentrative Nucleoside Transporters
ENTs	Equilibrative Nucleoside Transporters
FIC	Fractional Inhibitory Concentration
GEM	Gemcitabine
hCNT	Human nucleoside transporters
hOCT	Human organic cation transporters
IC_50_	The concentrations that inhibit cell growth by 50%
IHC	Immunohistochemistry
MDR	Multidrug resistance
MRP1	Multidrug resistance-associated protein 1
MTT	3-[4,5-dimethylthiazole-2-yl]-2,5-diphenyltetrazolium bromide
NQO1	NAD(P)H-quinone oxidoreductase-1
OCTs	Organic Cation Transporters
P-gp	P-glycoprotein
TET	Tetrandrine
