# Supplementary material for: Synergistic Effects of Atractylodes-Derived Sesquiterpenes and Polyacetylene on Chemotherapeutic Sensitivity in Cholangiocarcinoma: Impact on Transporter Gene Expression
Source: Molecules. 2026 Mar 29;31(7):1124. doi: 10.3390/molecules31071124 (PMC13074752; doi:10.3390/molecules31071124)
Supplement: Supplementary file 1 [file molecules-31-01124-s001.zip › molecules-4178164-supplementary.pdf]

Supplementary Information

Synergistic Effects of Atractylodes-Derived Sesquiterpenes and Polyacetylene on Chemotherapeutic Sensitivity in Cholangiocarcinoma: Impact on Transporter Gene Expression

Inthuon Kulma [1,2], Wanna Chaijaroenkul [1,2] and Kesara Na Bangchang [1,2,3,*]

[1] Graduate Program in Bioclinical Sciences, Chulabhorn International College of Medicine, Thammasat University (Rangsit Campus), Pathumthani 12120, Thailand; inthuorn.kulma@gmail.com (I.K.); wn_ap39@yahoo.com (W.C.)
[2] Center of Excellence in Pharmacology and Molecular Biology of Malaria and Cholangiocarcinoma, Thammasat University (Rangsit Campus), Pathumthani 12120, Thailand
[3] Drug Discovery and Development Center, Office of Advanced Science and Technology, Thammasat University (Rangsit Campus), Pathumthani 12120, Thailand
* Correspondence: kesaratmu@yahoo.com

Materials and Methods for Supplementary Figures **S1–S4**.

(1) Maintenance of human CCA cell lines. Three human CCA cell lines were utilized in this study. HuCCT1 and HUH28 were obtained from the Japanese Collection of Research Bioresources (JCRB) Cell Bank, National Institutes of Biomedical Innovation, Health and Nutrition (Ibaraki, Osaka, Japan). HuCCT1 was originally established from the malignant ascites of a patient with moderately differentiated adenocarcinoma of the intrahepatic bile duct tree, while HUH28 was established in vitro from a patient with cholangiocarcinoma of the intrahepatic bile duct tree. The CL-6 cell, originally isolated from the tumour tissue of a CCA patient at Siriraj Hospital (Mahidol University, Thailand), was kindly provided by Associate Professor Adisak Wongkajornslip. All cell lines were cultured in RPMI 1640 medium supplemented with 10% FBS, 100 U/mL penicillin, and 100 U/mL streptomycin, purchased from Gibco BRL Life Technologies (Grand Island, NY, USA). Cell cultures were maintained in a logarithmic phase at 37 °C in a humidified atmosphere containing 5% CO_2.

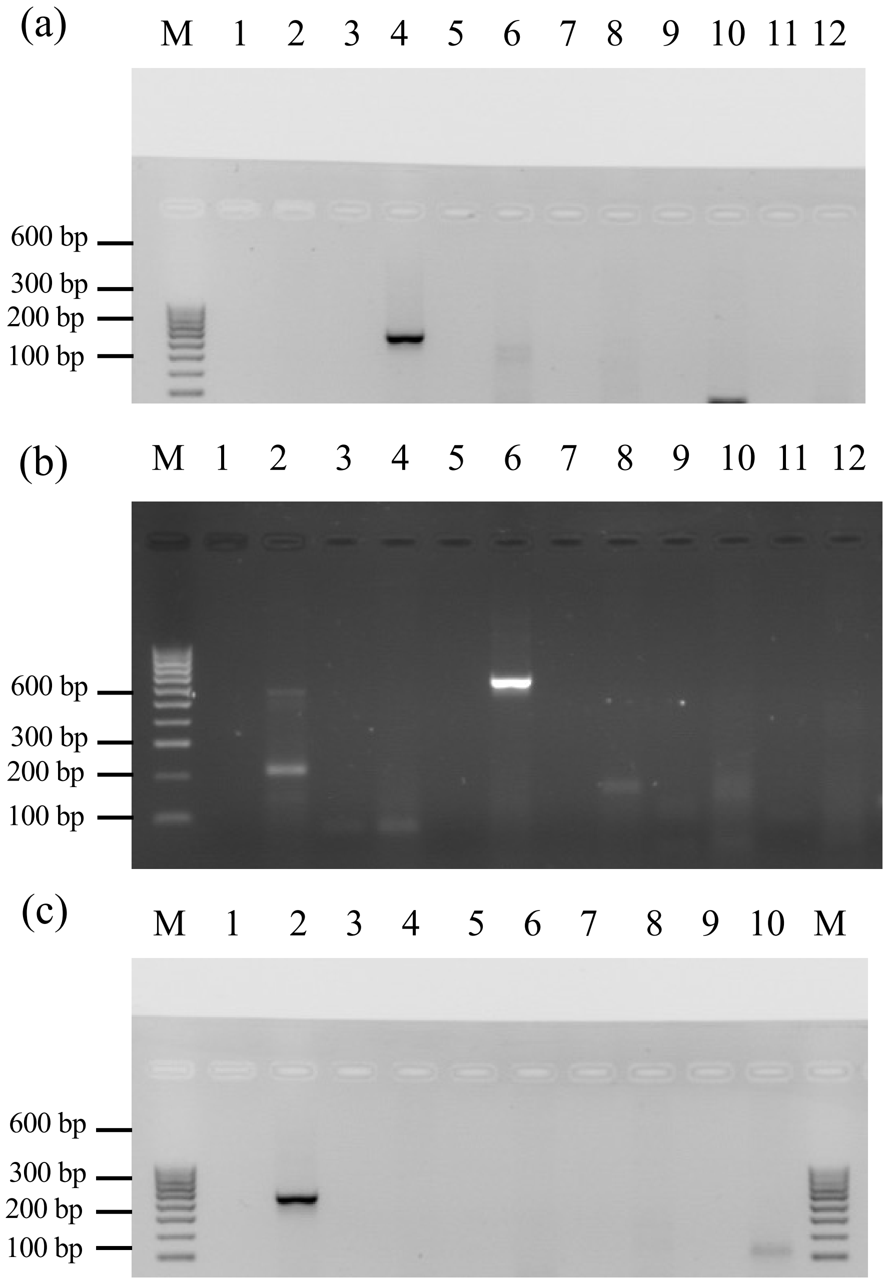

Supplementary Figure **S1**. Agarose gel showing the PCR products obtained from CL-6 cell lines with primers targeting the housekeeping gene and efflux transporter genes. **(a)** Lane M, 100 bp plus DNA marker; Lane 1&2, no template control and with template sample of *GAPDH*-328 bp; Lane 3&4, no template control and with template sample of *GAPDH*-628 bp; Lane 5&6, no template control and with template sample of *GAPDH*-100 bp; Lane 7&8, no template control and with template sample of *ABCB1*-206 bp; Lane 9&10, no template control and with template sample of *ABCC1*-262 bp; Lane 11&12, no template control and with template sample of *ABCC2*-202 bp. **(b)** Lane M, 100 bp plus DNA marker; Lane 1&2, no template control and with template sample of *GAPDH*-100 bp; Lane 3&4, no template control and with template sample of *GAPDH*-328 bp; Lane 5&6, no template control and with template sample of *GAPDH*-628 bp; Lane 7&8, no template control and with template sample of *ABCC3*-156 bp;

Lane 9&10, no template control and with template sample of *ABCC4*-143 bp; Lane 11&12, no template control and with template sample of *ABCC11*-242 bp. **(c)** Lane M, 100 bp plus DNA marker; Lane 1&2, no template control and with template sample of *GAPDH*-628 bp; Lane 3&4, no template control and with template sample of *ABCC2*-202 bp; Lane 5&6, no template control and with template sample of *ABCC4*-143 bp; Lane 7&8, no template control and with template sample of *ABCC11*-242 bp; Lane 9&10, no template control and with template sample of *ABCG2*-206 bp.

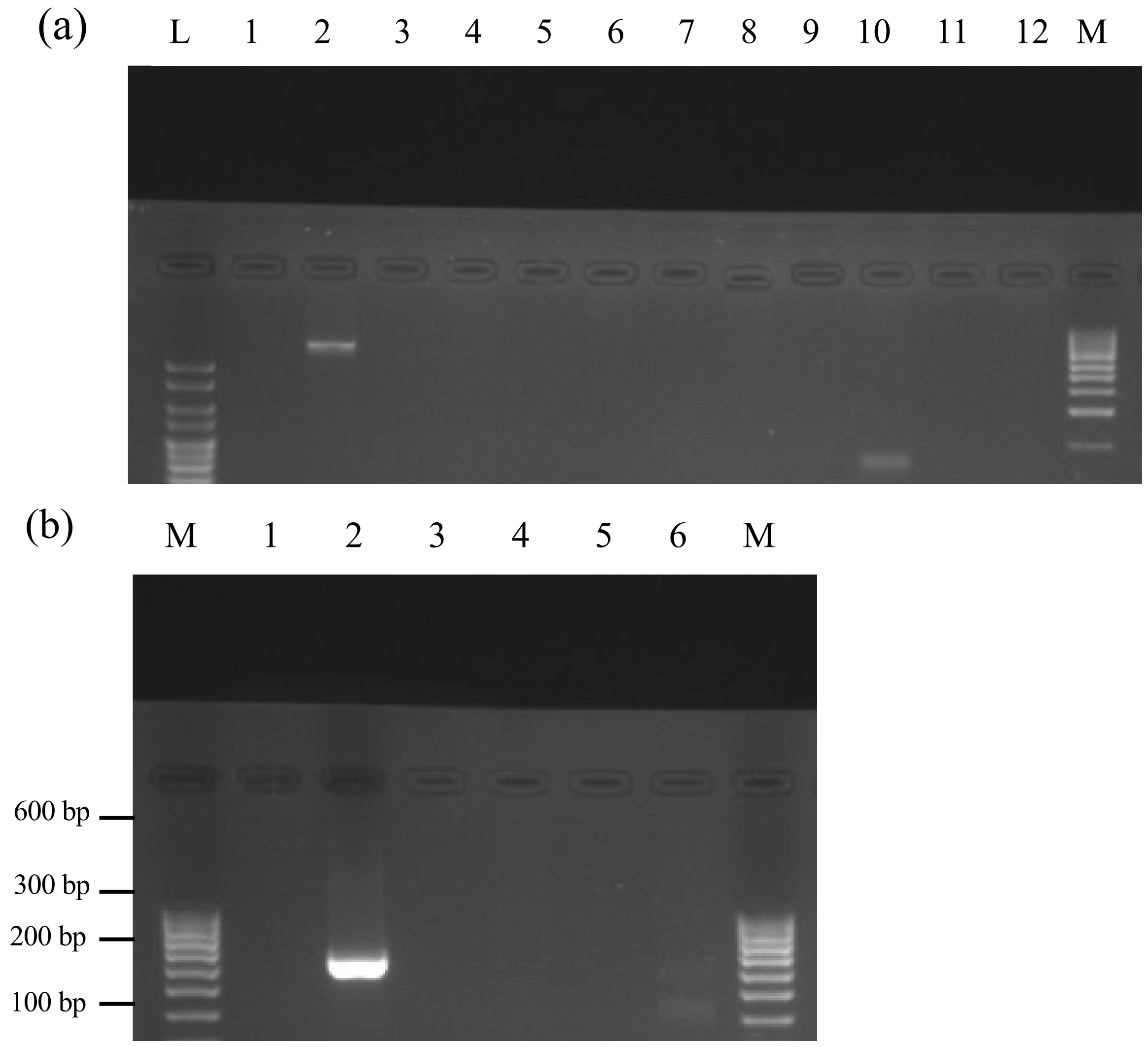

Supplementary Figure **S2**. Agarose gel showing the PCR products obtained from CL-6 cell lines with primer targeting housekeeping and reuptake transporter genes. **(a)** Lane L, 25 bp plus DNA marker; Lane M, 100 bp plus DNA marker; Lane 1&2, no template control and with template sample of *GAPDH*-628 bp; Lane 3&4, no template control and with template sample of *hCNT1*-75 bp; Lane 5&6, no template control and with template sample of *hCNT2*-75 bp; Lane 7&8, no template control and with template sample of *hCNT3*-90 bp; Lane 9&10, no template control and with template sample of *hENT1*-151 bp; Lane 11&12, no template control and with template sample of *hENT2*-81 bp. **(b)** Lane M, 100 bp plus DNA marker; Lane 1&2, no template control and with template sample of GAPDH-628 bp; Lane 3&4, no template control and with template sample of *hOCT1*-368 bp; Lane 5&6, no template control and with template sample of *hOCT3*-324 bp.

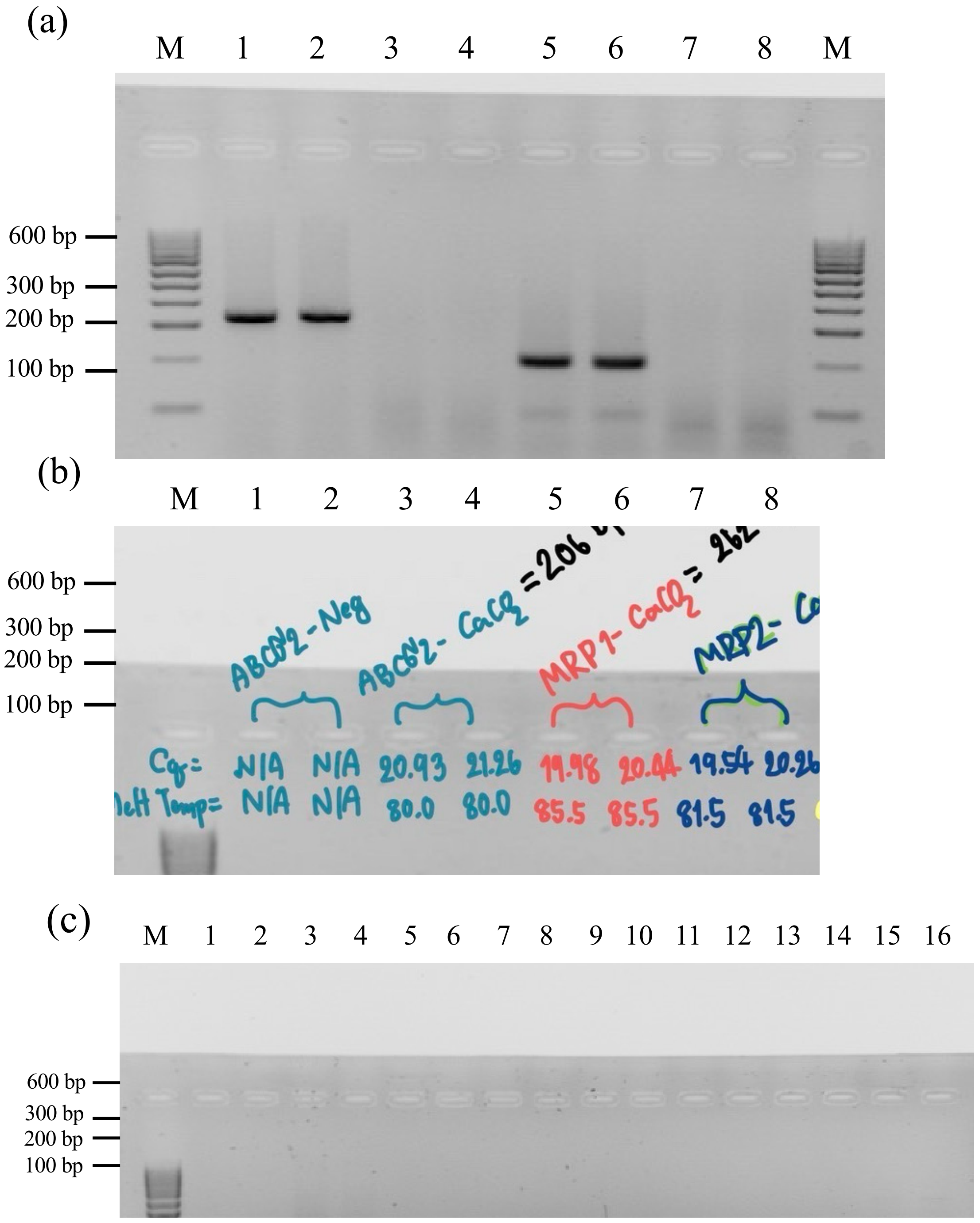

Supplementary Figure **S3**. Agarose gel showing the PCR products obtained from Caco2 cell lines (as a positive control) with primers targeting housekeeping and efflux transporter genes. **(a)** Lane M, 100 bp plus DNA marker; Lane 1&2, *GAPDH*-328 bp; Lane 3&4, negative control for *GAPDH*; Lane 5&6, *ABCB1*-206 bp; Lane 7&8, negative control for *ABCB1*. **(b)** Lane M, 100 bp plus DNA marker; Lane 1&2, negative control; Lane 3&4, *ABCG2*-206 bp; Lane 5&6, *ABCC1*-262 bp; Lane 7&8, *ABCC2*-202 bp. **(c)** Lane M, 100 bp plus DNA marker; Lane 1&2, negative control for *GAPDH*; Lane 3&4, *GAPDH*-328 bp; Lane 5&6, negative control for *ABCC3*; Lane 7&8, *ABCC3*-156 bp; Lane 9&10, negative control for *ABCC4*; Lane 11&12, *ABCC4*-143 bp; Lane 13&14, negative control for *ABCC11*; lane 15&16, *ABCC11*-242 bp.

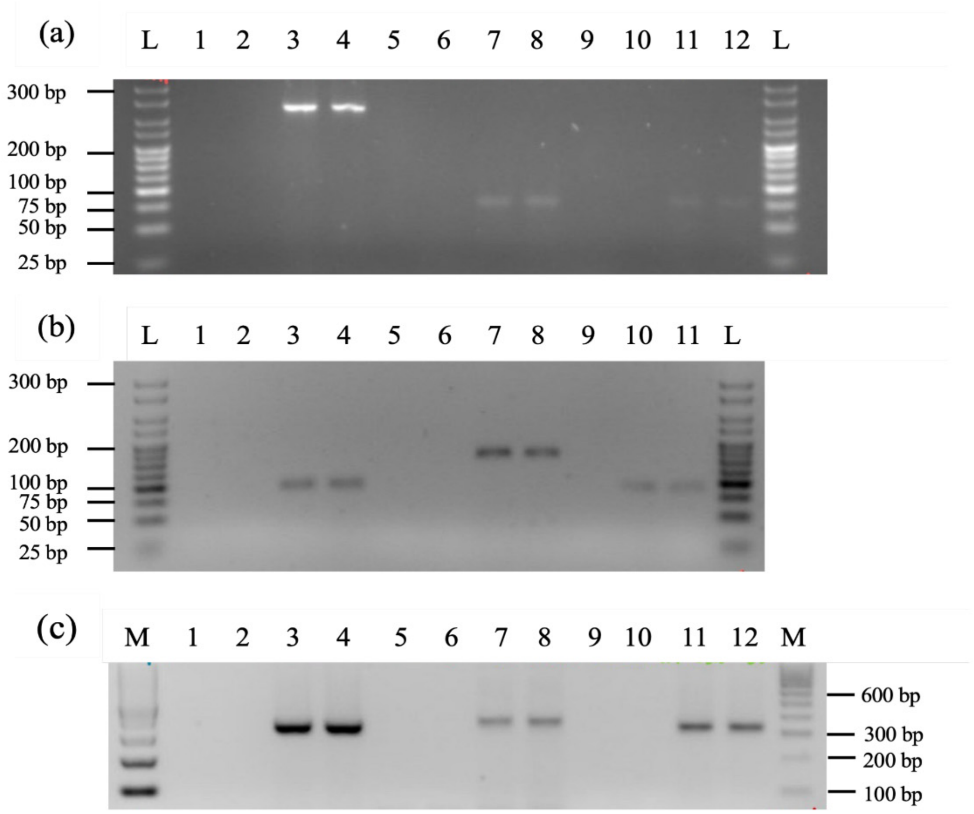

(a)
L 1 2 3 4 5 6 7 8 9 10 11 12 L
300 bp
200 bp
100 bp
75 bp
50 bp
25 bp
(b)
L 1 2 3 4 5 6 7 8 9 10 11 L
300 bp
200 bp
100 bp
75 bp
50 bp
25 bp
(c)
M 1 2 3 4 5 6 7 8 9 10 11 12 M
600 bp
300 bp
200 bp
100 bp

Supplementary Figure **S4**. Agarose gel showing the PCR products obtained from Caco2 cell lines (as a positive control) with primers targeting housekeeping and re-uptake transporter genes. **(a)** Lane L, 25 bp plus DNA marker; Lane 1&2, negative control for *GAPDH*; Lane 3&4, *GAPDH*-328 bp; Lane 5&6, negative control for *hCNT1*; Lane 7&8, *hCNT1*-75 bp, Lane 9&10, negative control for *hCNT2*; Lane 11&12, *hCNT2*-75 bp. **(b)** Lane L, 25 bp plus DNA marker; Lane 1&2, negative control for *hCNT3*; Lane 3&4, *hCNT3*-90 bp; Lane 5&6, negative control for *hENT1*; Lane 7&8, *hENT1*-151 bp, Lane 9, negative control for *hENT2*; Lane 10&11, *hENT2*-81 bp. **(c)** Lane M, 100 bp plus DNA marker; Lane 1&2, negative control for *GAPDH*; Lane 3&4, *GAPDH*-328 bp; Lane 5&6, negative control for *hOCT1*; Lane 7&8, *hOCT1*-363 bp; Lane 9&10, negative control for *hOCT3*; Lane 11&12, *hOCT3*-324 bp.

Results for Supplementary Figures **S5–S11** and Tables **S1** and **S2**.

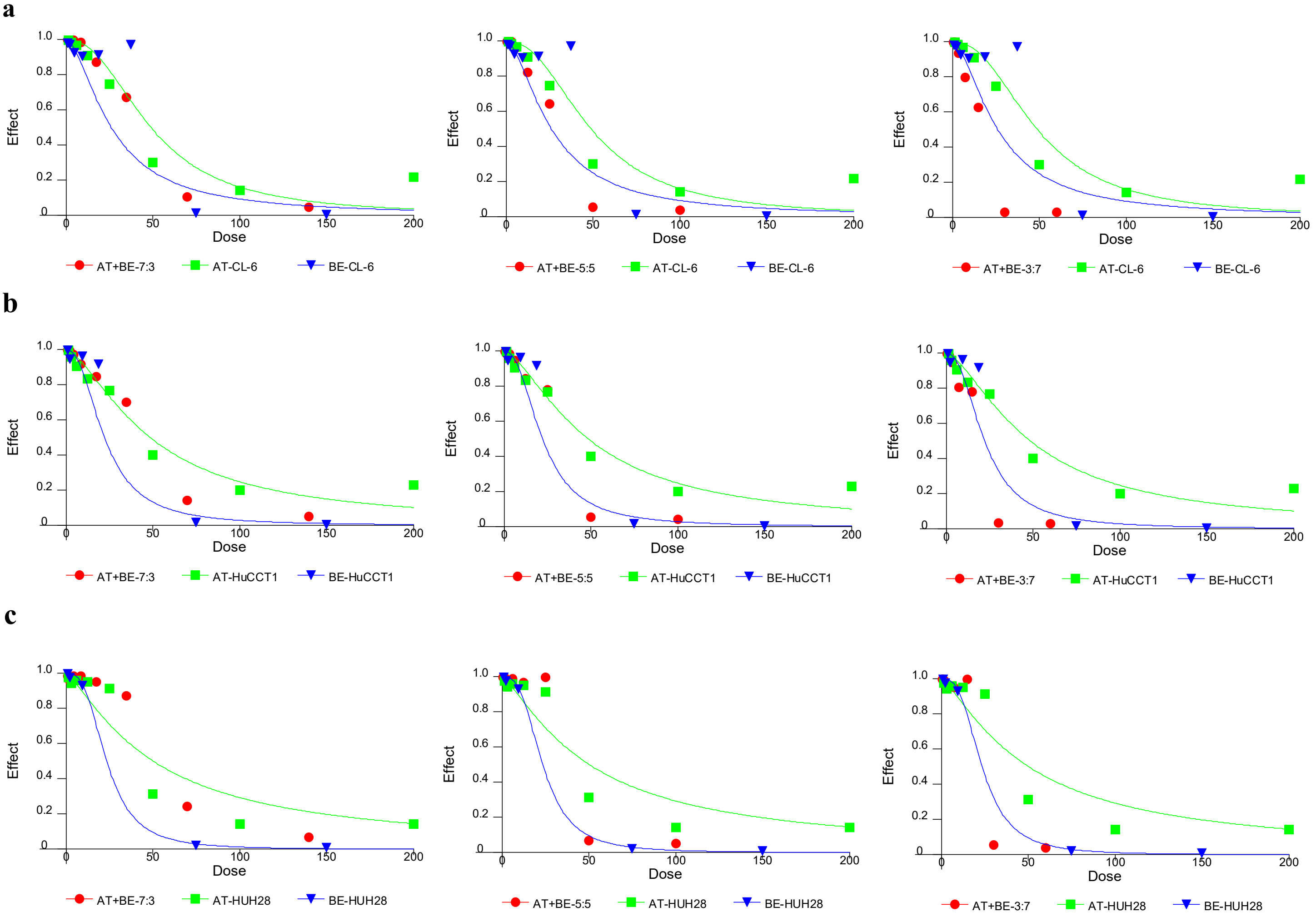

Supplementary Figure **S5**. Dose-response curves in CL-6 (a), HuCCT1 (b), and HuH28 (c) cell lines combining atractylodin (AT) and β-eudesmol (BE) at ratios of 7:3, 5:5, and 3:7 for 48 h. Results are presented as median from three replications.

Supplementary Figure **S6**. Dose-response curves in CL-6 (a), HuCCT1 (b), and HuH28 (c) cell lines combining atractylodin (AT) and cisplatin (CIS) at ratios of 7:3, 5:5, and 3:7 for 48 h. Results are presented as median from three replications.

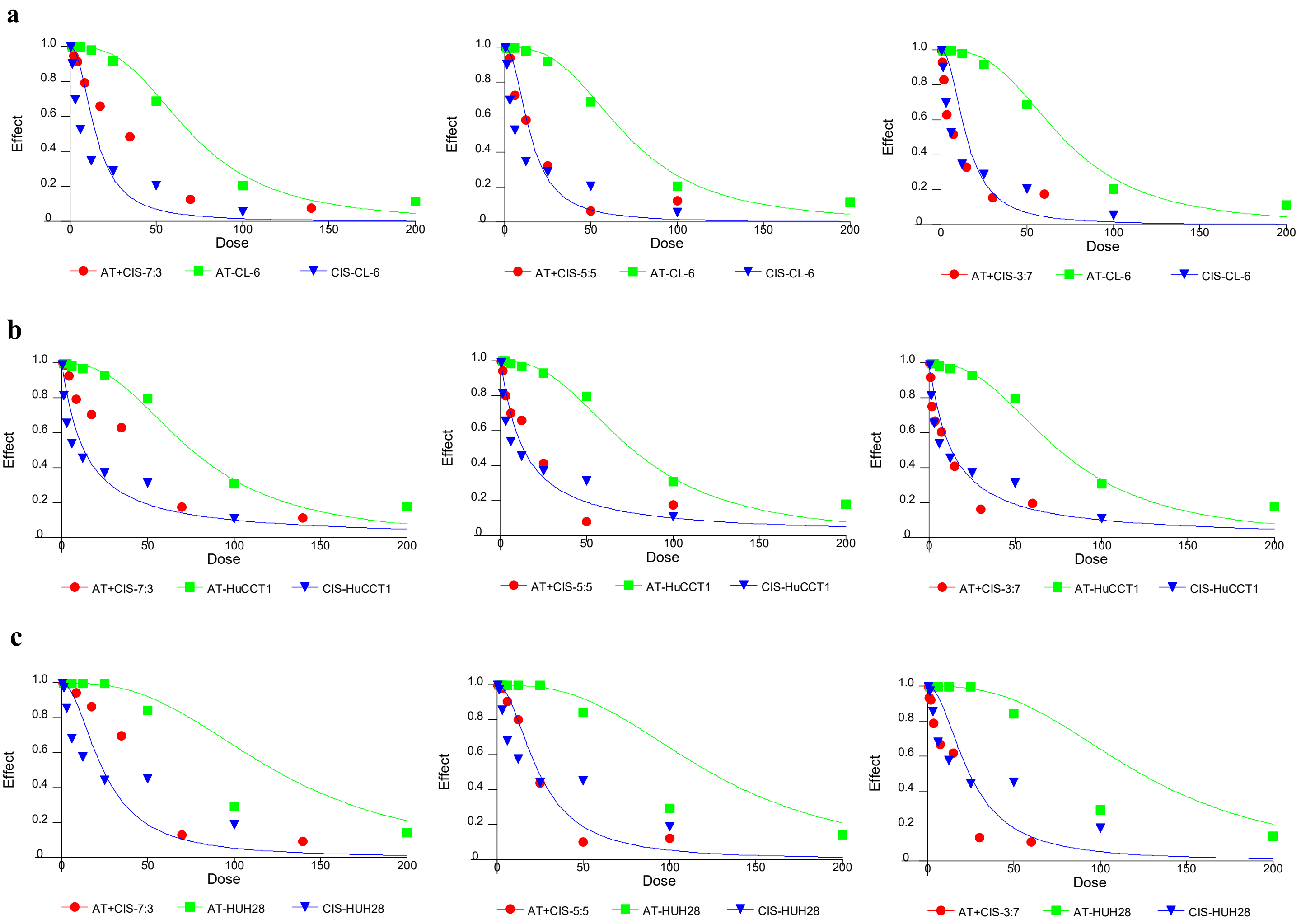

Supplementary Figure **S7**. Dose-response curves in CL-6 (a), HuCCT1 (b), and HuH28 (c) cell lines combining β-eudesmol (BE) and cisplatin (CIS) at ratios of 7:3, 5:5, and 3:7 for 48 h. Results are expressed as the median from three replications.

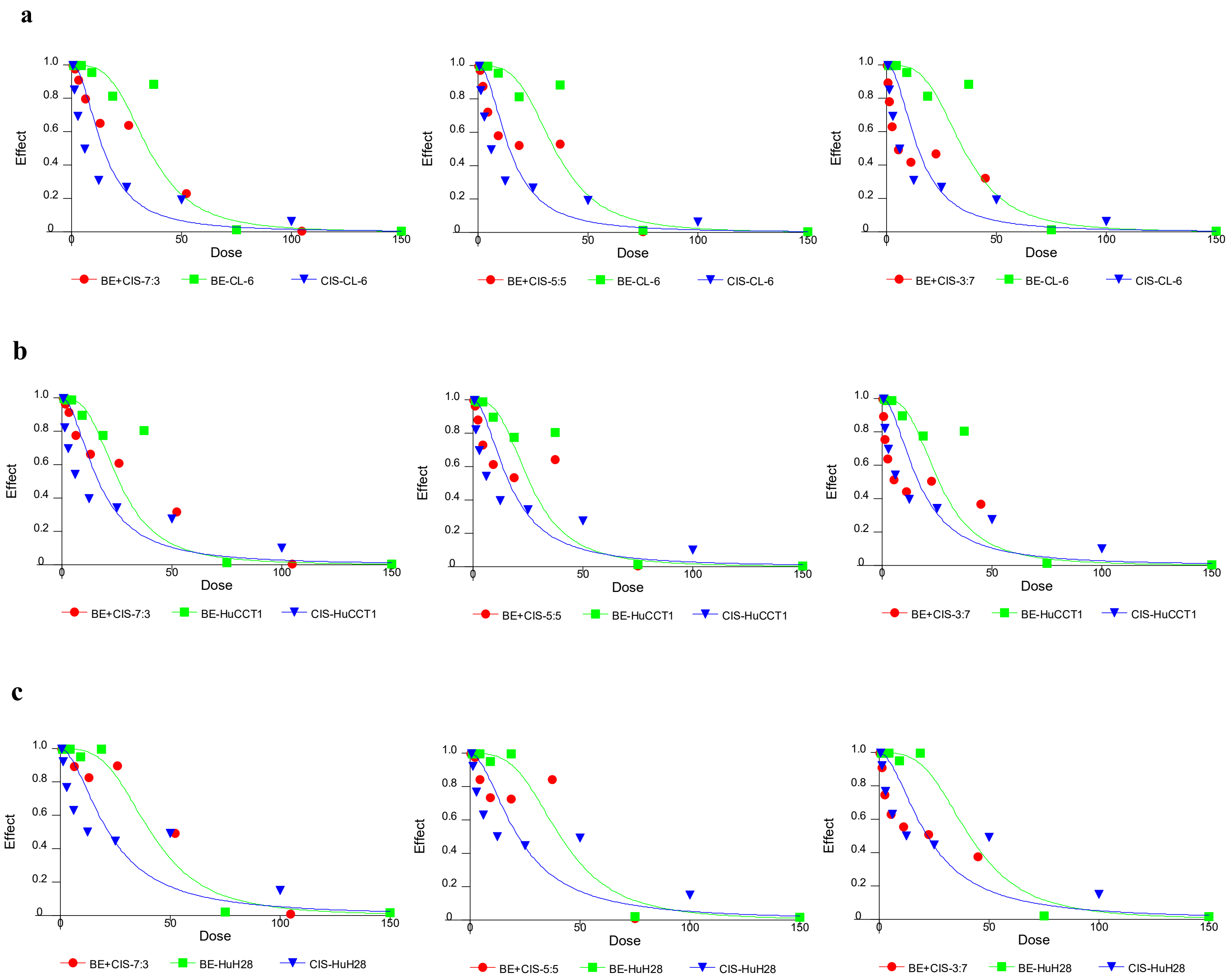

Supplementary Figure **S8**. Dose-response curves in CL-6 (a), HuCCT1 (b), and HuH28 (c) cell lines combining atractylodin (AT) and 5-fluorouracil (5-FU) at ratios of 7:3, 5:5, and 3:7 for 48 h. Results are presented as median from three replications.

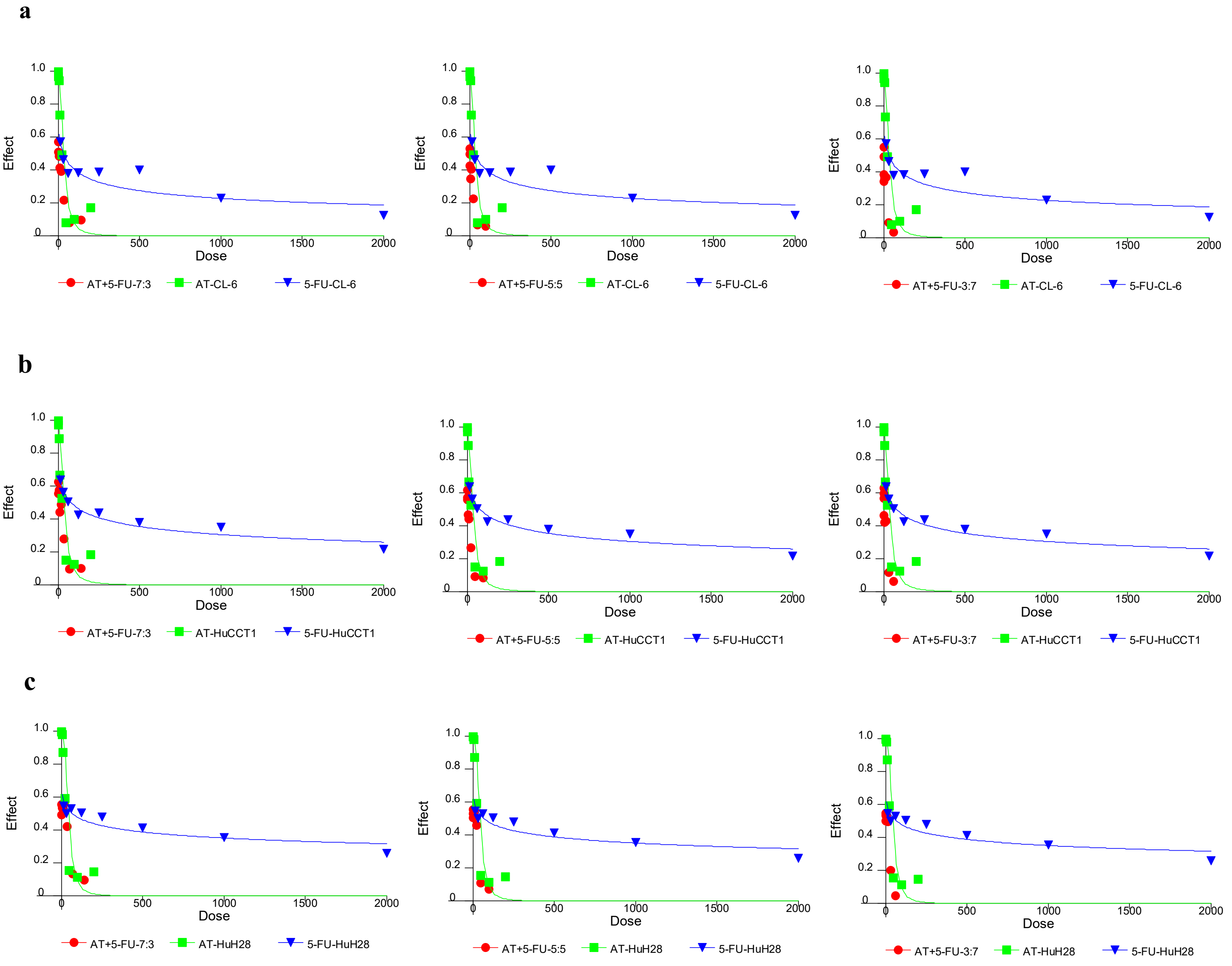

Supplementary Figure **S9**. Dose-response curves in CL-6 (a), HuCCT1 (b), and HuH28 (c) cell lines combining β-eudesmol (BE) and 5-fluorouracil (5-FU) at ratios of 7:3, 5:5, and 3:7 for 48 h. Results are presented as median from three replications.

a

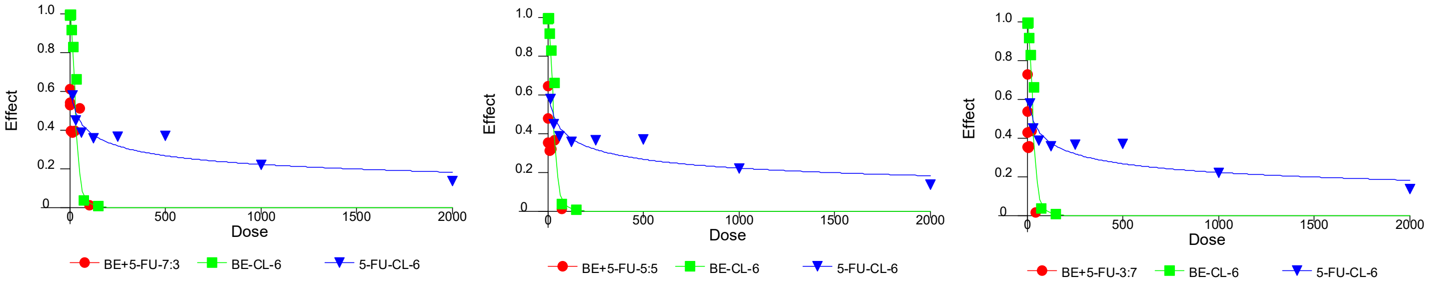

b

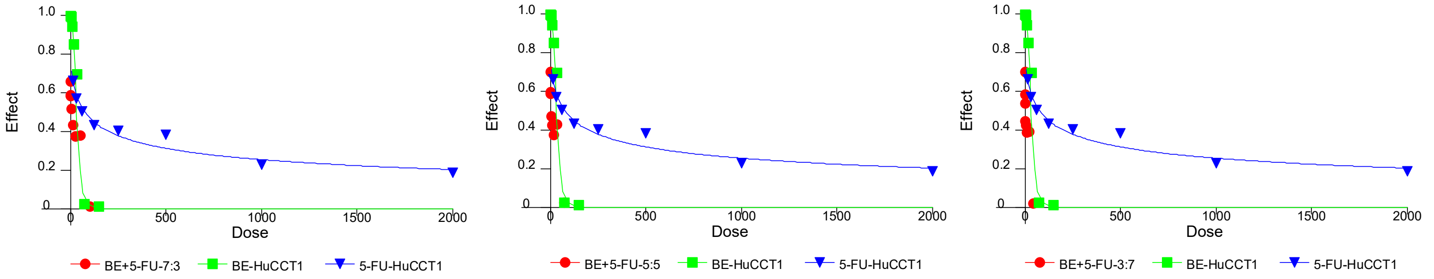

c

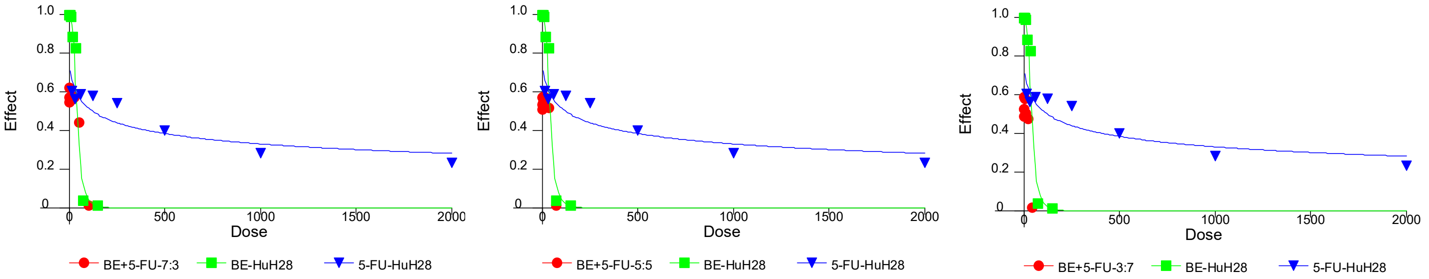

Supplementary Figure **S10**. Dose-response curves in CL-6 (a), HuCCT1 (b), and HuH28 (c) cell lines combining atractylodin (AT) and gemcitabine (GEM) at ratios of 7:3, 5:5, and 3:7 for 48 h. Results are presented as median from three replications.

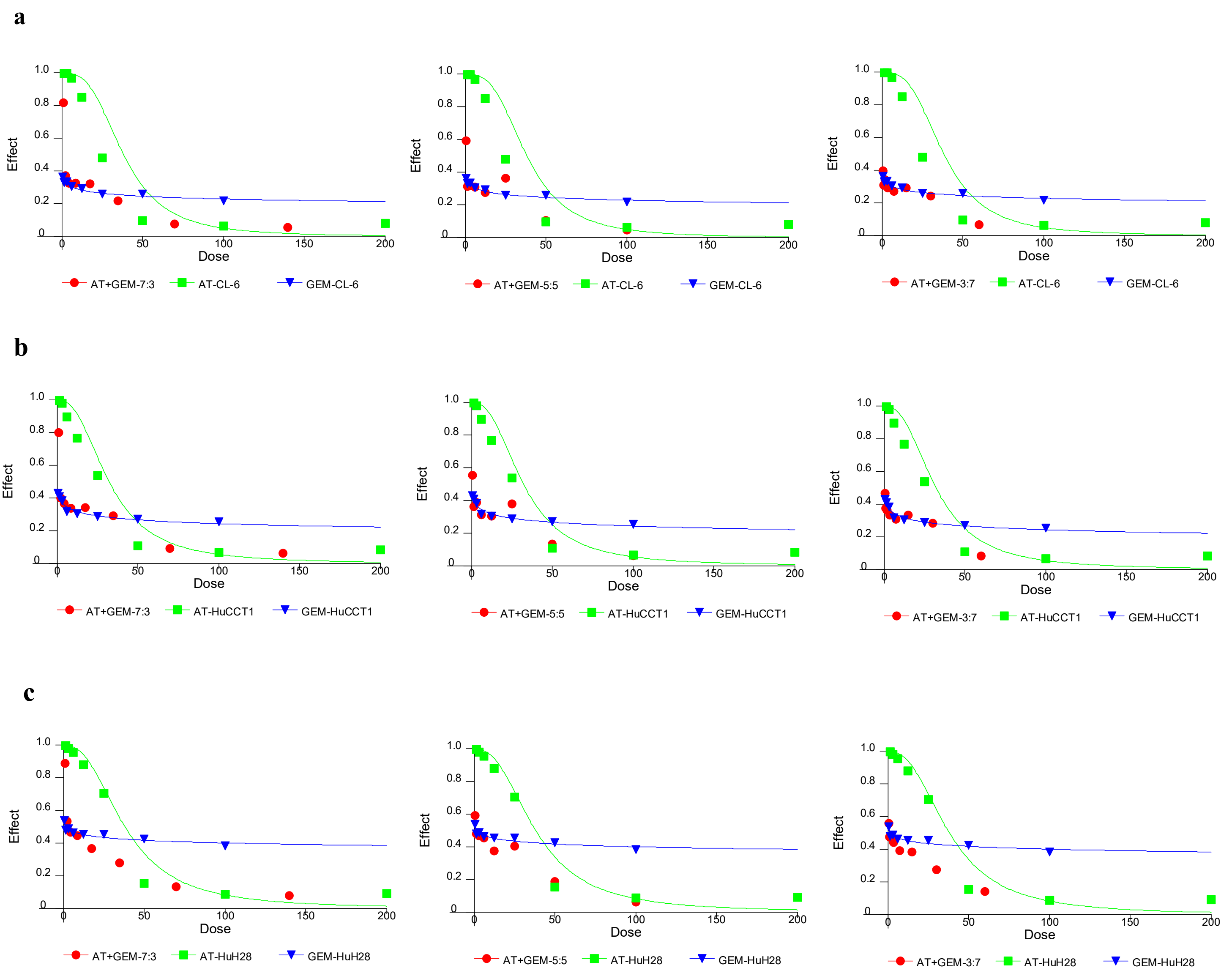

Supplementary Figure **S11**. Dose-response curves in CL-6 (a), HuCCT1 (b), and HuH28 (c) cell lines combining β-eudesmol (BE) and gemcitabine (GEM) at ratios of 7:3, 5:5, and 3:7 for 48 h. Results are presented as median from three replications.

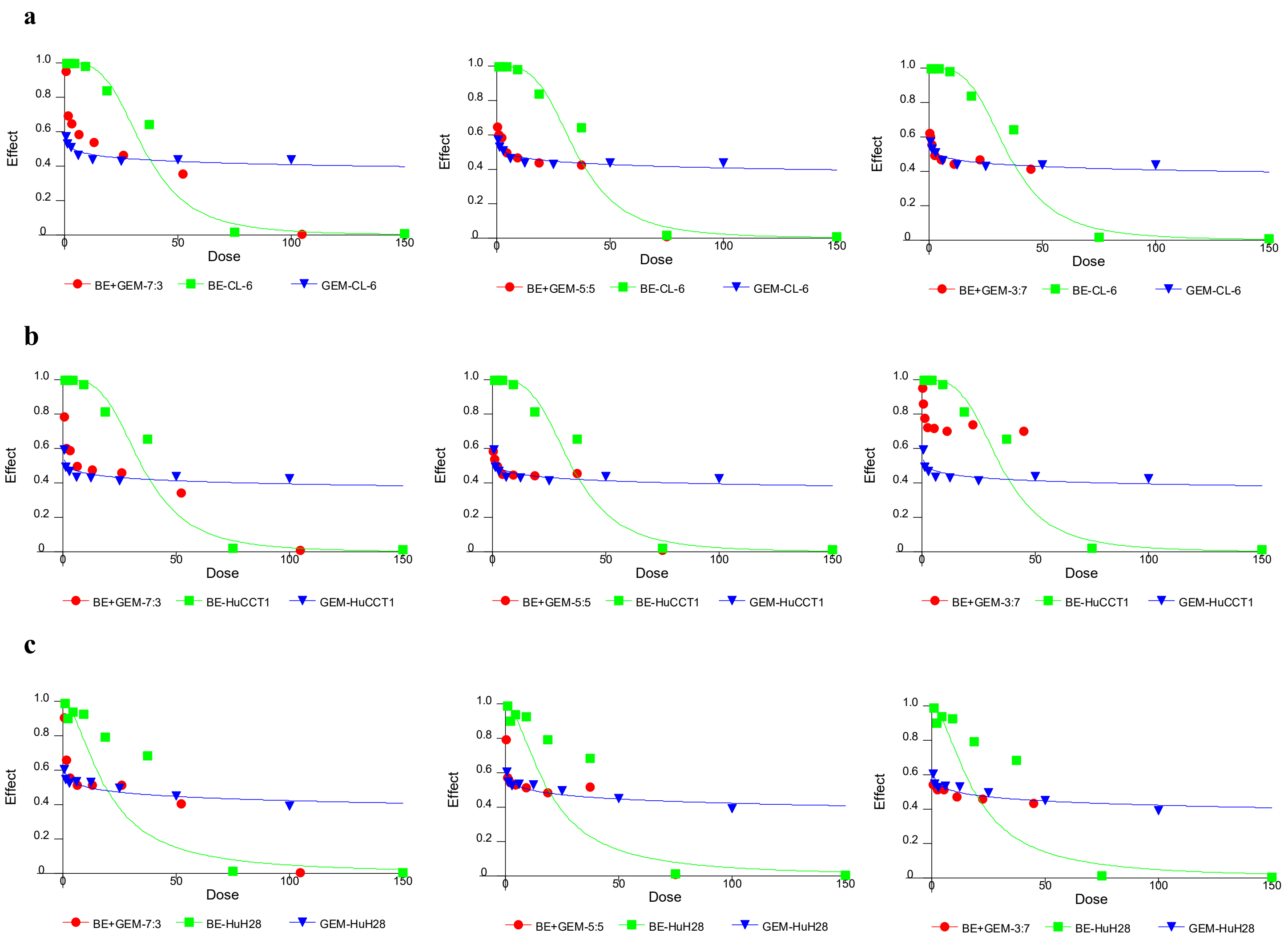

Supplementary Table **S1**. Relative mRNA expression, compared to *GAPDH* expression, of the efflux transporters in CL6 cell line following exposure to atractylodin (AT) and β-eudesmol (BE) alongside standard chemotherapies for CL-6, including 5-FU, cisplatin, and gemcitabine. Data are presented as median (range) values. Statistical significance was determined with the Mann-Whitney U test (Statistically significant difference with untreated control cells, * p=0.03, statistically significant difference between single and combination treatment, ** *p=0.05*).

Genes	Control	AT alone	BE alone	AT + BE
MDR1	1	0.399 (0.298-0.616)*	1.147 (0.691-1.728)	1.361 (1.024-1.459)*
MRP1		1.580 (1.384-1.858)	2.924 (0.551-3.106)	3.034 (2.404-3.674)
MRP2		0.279 (0.111-0.376)*	0.538 (0.431-0.625)*	0.696 (0.376-0.896)*
MRP3		0.199 (0.098-1.297)	1.974 (1.422-3.151)	2.153 (1.930-2.887)
MRP4		0.751 (0.553-1.196)**	1.443 (0.712-3.991)	2.426 (1.436-3.131)*
MRP11		0.392 (0.334-0.451)*, **	0.703 (0.630-1.189)	0.717 (0.634-1.017)
BCRP		0.352 (0.295-0.543)*, **	0.790 (0.002-1.038)**	1.393 (1.276-2.412)*
		**AT alone**	**CIS alone**	**AT + CIS**
MDR1	1	0.399 (0.298-0.616)*, **	0.842 (0.739-1.556)	1.512 (1.388-2.012)*
MRP1		1.580 (1.384-1.858)	0.430 (0.260-1.988)	1.039 (0.464-1.074)
MRP2		0.279 (0.111-0.376)*, **	0.527 (0.325-0.852)*, **	2.234 (1.890-2.885)*
MRP3		0.199 (0.098-1.297)	1.378 (0.329-5.077)	0.000
MRP4		0.751 (0.553-1.196)**	5.735 (0.869-10.084)	12.895 (6.716-16.015)*
MRP11		0.392 (0.334-0.451)*, **	0.883 (0.759-1.098)**	2.657 (2.122-4.274)*
BCRP		0.352 (0.295-0.543)*, **	1.866 (0.840-3.209)**	6.170 (5.193-9.927)*
		**BE alone**	**CIS alone**	**BE + CIS**
MDR1	1	1.147 (0.691-1.728)	0.842 (0.739-1.556)	0.762 (0.556-0.800)*
MRP1		2.924 (0.551-3.106)	0.430 (0.260-1.988)	0.185 (0.152-2.792)
MRP2		0.538 (0.431-0.625)*	0.527 (0.325-0.852)*	0.179 (0.082-0.774)*
MRP3		1.974 (1.422-3.151)	1.378 (0.329-5.077)	3.008 (1.029-5.344)
MRP4		1.443 (0.712-3.991)	5.735 (0.869-10.084)	2.378 (1.366-3.871)*
MRP11		0.703 (0.630-1.189)	0.883 (0.759-1.098)**	0.584 (0.003-0.700)*
BCRP		0.790 (0.002-1.038)	1.866 (0.840-3.209)**	0.656 (0.296-0.798)*
		**AT alone**	**5-FU alone**	**AT + 5-FU**
MDR1	1	0.399 (0.298-0.616)*, **	0.105 (0.064-0.145)*, **	0.918 (0.680-1.045)
MRP1		1.580 (1.384-1.858)	2.135 (2.051-2.243)	0.754 (0.742-0.796)
MRP2		0.279 (0.111-0.376)*, **	0.602 (0.178-0.842)*	0.874 (0.560-0.984)*
MRP3		0.199 (0.098-1.297)	1.264 (0.329-5.077)	1.044 (0.633-2.182)
MRP4		0.751 (0.553-1.196)**	5.543 (2.916-8.372)*	4.195 (3.961-6.798)*
MRP11		0.392 (0.334-0.451)*, **	1.166 (1.112-1.363)*	1.502 (1.136-1.570)*
BCRP		0.352 (0.295-0.543)*, **	0.914 (0.006-2.697)	2.621 (2.265-3.367)*

Supplementary Table **S1**. Relative mRNA expression, compared to GAPDH expression, of the efflux transporters in CL6 cell line following exposure to atractylodin (AT) and β-eudesmol (BE) alongside standard chemotherapies for CL-6, including 5-FU, cisplatin, and gemcitabine. Data are presented as median (range) values. Statistical significance was determined using the Mann-Whitney U test (statistically significant difference with untreated control cells, * p=0.03; statistically significant difference between single and combination treatment, ** *p=0.05*). (Cont.)

**Genes**	**Control**	**BE alone**	**5-FU alone**	**BE + 5-FU**
MDR1	1	1.147 (0.691-1.728)	0.105 (0.064-0.145)*, **	1.057 (0.825-1.147)
MRP1		2.924 (0.551-3.106)	2.135 (2.051-2.243)	1.124 (1.071-1.432)
MRP2		0.538 (0.431-0.625)*, **	0.602 (0.178-0.842)*	0.337 (0.103-0.373)*
MRP3		1.974 (1.422-3.151)	1.264 (0.329-5.077)	2.637 (2.124-2.950)
MRP4		1.443 (0.712-3.991)**	5.543 (2.916-8.372)*	7.505 (6.745-22.010)*
MRP11		0.703 (0.630-1.189)	1.166 (1.112-1.363)*	0.992 (0.657-1.287)
BCRP		0.790 (0.002-1.038)**	0.914 (0.006-2.697)	1.416 (1.121-1.966)*
		**AT alone**	**GEM alone**	**AT + GEM**
MDR1	1	0.399 (0.298-0.616)*, **	2.546 (1.819-2.725)*, **	4.646 (4.402-5.720)*
MRP1		1.580 (1.384-1.858)	1.186 (1.128-1.679)	1.767 (1.113-1.918)
MRP2		0.279 (0.111-0.376)*, **	1.839 (1.445-3.086)*, **	6.811 (5.705-7.907)*
MRP3		0.199 (0.098-1.297)	0.000	0.000
MRP4		0.751 (0.553-1.196)**	2.023 (1.935-5.496)*, **	24.394 (15.971-37.460)*
MRP11		0.392 (0.334-0.451)*, **	2.126 (1.919-2.356)*, **	8.408 (8.402-8.492)*
BCRP		0.352 (0.295-0.543)*, **	4.270 (3.488-5.010)*, **	24.817 (16.540-39.473)*
		**BE alone**	**GEM alone**	**BE + GEM**
MDR1	1	1.147 (0.691-1.728)	2.546 (1.819-2.725)*	1.841 (1.573-2.059)*
MRP1		2.924 (0.551-3.106)	1.186 (1.128-1.679)	2.647 (2.630-3.111)
MRP2		0.538 (0.431-0.625)*, **	1.839 (1.445-3.086)*	1.201 (1.028-1.752)*
MRP3		1.974 (1.422-3.151)	0.000	2.208 (1.033-5.333)
MRP4		1.443 (0.712-3.991)**	2.023 (1.935-5.496)*	9.604 (4.268-13.983)*
MRP11		0.703 (0.630-1.189)	2.126 (1.919-2.356)*	1.088 (0.885-3.361)
BCRP		0.790 (0.002-1.038)**	4.270 (3.488-5.010)*	3.806 (2.831-5.097)*

Supplementary Table **S2**. Relative mRNA expression, compared to GAPDH expression, of the uptake transporters in CL6 cell line following exposure to atractylodin (AT) and β-eudesmol (BE) alongside standard chemotherapies for CL-6, including 5-FU, cisplatin, and gemcitabine. Data are presented as median (range) values. Statistical significance was determined using the Mann-Whitney U test (statistically significant difference with untreated control cells, * p=0.03; statistically significant difference between single and combination treatment, ** *p=0.05*).

**Genes**	**Control**	**AT alone**	**BE alone**	**AT + BE**
hENT1	1	1.652 (1.042-4.664)*	2.751 (0.614-3.646)	2.648 (0.599-4.239)
hOCT1		0.438 (0.218-0.533)*	0.057 (0.048-0.268)*, **	0.604 (0.270-1.077)*
hOCT3		1.424 (1.137-1.828)*	0.555 (0.053-5.567)	3.045 (1.078-4.044)*
		**AT alone**	**CIS alone**	**AT + CIS**
hENT1	1	1.652 (1.042-4.664)*, **	2.625 (1.493-3.699)*, **	13.873 (8.645-21.625)*
hOCT1		0.438 (0.218-0.533)*, **	1.004 (0.529-1.187)	2.900 (1.051-3.518)*
hOCT3		1.424 (1.137-1.828)*, **	1.430 (0.926-3.613)**	18.842 (7.657-57.801)*
		**BE alone**	**CIS alone**	**BE + CIS**
hENT1	1	2.751 (0.614-3.646)	2.625 (1.493-3.699)*	3.318 (1.009-4.096)*
hOCT1		0.057 (0.048-0.268)*	1.004 (0.529-1.187)	0.306 (0.003-0.970)*
hOCT3		0.555 (0.053-5.567)	1.430 (0.926-3.613)**	0.539 (0.363-0.675)*
		**AT alone**	**5-FU alone**	**AT + 5-FU**
hENT1	1	1.652 (1.042-4.664)*	4.644 (1.396-9.631)*	2.641 (1.567-5.279)*
hOCT1		0.438 (0.218-0.533)*	0.228 (0.037-0.781)*	0.908 (0.386-1.570)
hOCT3		1.424 (1.137-1.828)*, **	1.083 (0.437-5.312)	5.028 (1.954-8.895)*
		**BE alone**	**5-FU alone**	**BE + 5-FU**
hENT1	1	2.751 (0.614-3.646)	4.644 (1.396-9.631)*	3.126 (0.862-43.049)
hOCT1		0.057 (0.048-0.268)*	0.228 (0.037-0.781)*	0.365 (0.157-0.383)*
hOCT3		0.555 (0.053-5.567)	1.083 (0.437-5.312)	0.175 (0.045-6.175)
		**AT alone**	**GEM alone**	**AT + GEM**
hENT1	1	1.652 (1.042-4.664)*, **	5.983 (5.535-6.248)*, **	33.830 (24.653-39.969)*
hOCT1		0.438 (0.218-0.533)*, **	1.482 (1.085-2.386)*, **	9.700 (5.817-10.760)*
hOCT3		1.424 (1.137-1.828)*, **	3.342 (1.661-4.462)*, **	43.454 (31.757-147-923)*
		**BE alone**	**GEM alone**	**BE + GEM**
hENT1	1	2.751 (0.614-3.646)	5.983 (5.535-6.248)*	3.765 (2.969-8.882)*
hOCT1		0.057 (0.048-0.268)*, **	1.482 (1.085-2.386)*	2.553 (1.807-4.076)*
hOCT3		0.555 (0.053-5.567)	3.342 (1.661-4.462)*	5.150 (3.592-39.041)*
